# Engineered Chiral Probiotics Ameliorate MASH via Gut–Liver Axis

**DOI:** 10.1002/advs.78432

**Published:** 2026-11-04

**Authors:** Jing Chen, Shen Zhang, Yuli Zhu, Da Zhou, Chunya Ji, Mingrui Zhu, Sirui Wang, Yuting Shen, Jifeng Yu, Heng Zhang, Yan Fang, Huixiong Xu, Haohao Yin

**Affiliations:** ^1^ Department of Ultrasound Zhongshan Hospital Institute of Ultrasound in Medicine and Engineering Fudan University Shanghai People's Republic of China; ^2^ Department of Ultrasound Zhuhai People's Hospital (The Affiliated Hospital of Beijing Institute of Technology Zhuhai Clinical Medical College of Jinan University) Zhuhai People's Republic of China; ^3^ Department of Gastroenterology Zhongshan Hospital Fudan University Shanghai People's Republic of China; ^4^ Department of Ultrasound Huashan Hospital Fudan University Shanghai People's Republic of China

**Keywords:** biology, dysbiosis, fibrosis, inflammation, kynurenic acid, linoleic acid, microbiome, probiotic, steatohepatiti, steatosis

## Abstract

The global epidemic of metabolic dysfunction‐associated steatohepatitis (MASH) is escalating without effective therapies. This study identified a significant depletion of the gut commensal bacterium *Faecalibacterium duncaniae* (*F. duncaniae*) as a core feature of MASH. To overcome the poor colonization by traditional probiotics in inflammatory environments, we engineered a living microbial therapeutic by incorporating chiral gold nanozymes onto *F. duncaniae*. We discovered that biological performance is enantiomer‐dependent: the right‐handed nanozyme (D‐Au) serves as a superior ROS‐scavenging shield, protecting the probiotic from oxidative assault and enhancing its colonization in the inflamed gut. In mouse models of MASH, this engineered probiotic (F@D‐Au) reversed disease progression, ameliorating steatosis, inflammation, and fibrosis. It further restored systemic homeostasis, rectifying dysbiosis, metabolic disturbances, and—notably—MASH‐associated cognitive impairment. Metabolomic analyses revealed that this multi‐organ benefit was linked to the restoration of key microbial metabolites, including kynurenic acid and linoleic acid. Our work establishes a new class of chirality‐enhanced living therapeutics that precisely remodel the gut–liver axis, offering a transformative strategy for treating MASH and its complications.

## Graphical abstract

A chiral gold nanozyme was coated onto the gut commensal bacterium *Faecalibacterium duncaniae (F. duncaniae)* to create an engineered probiotic. In mouse models of MASH, this orally administered living therapeutic protected the bacterium against oxidative stress, enhanced its gut colonization, reversed steatosis, inflammation, and fibrosis, and improved dysbiosis, metabolic disturbances, and cognitive impairment.

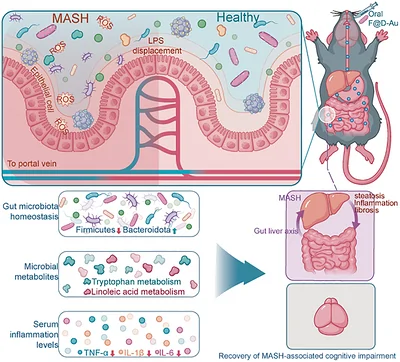

## 1 Introduction

The rising prevalence of metabolic syndrome has established metabolic dysfunction‐associated steatotic liver disease (MASLD) as the most common chronic liver condition globally. Its advanced form, MASH, which can progress to cirrhosis and liver cancer [1], is projected to become the leading indication for liver transplantation [2]. This looming crisis is met with a stark therapeutic void; effective treatment options for MASH remain limited [3, 4]. The recent approval of Resmetirom, while a milestone, underscores the paucity of options and the urgent need for mechanistically distinct approaches [5]. Therefore, the development of novel therapeutic strategies is urgently needed.

An emerging paradigm posits the gut–liver axis as a central driver of MASH [6, 7, 8]. Disruption of the gut microbiome can impair intestinal barrier function, promoting gut‐derived inflammatory signals that are transmitted to the liver, thereby exacerbating hepatic inflammation [9]. In the context of chronic metabolic diseases, persistent liver inflammation is often accompanied by a systemic low‐grade inflammatory state, which is closely associated with abnormalities in multiple systems. Therefore, restoring gut homeostasis and alleviating gut–liver inflammatory signaling are considered promising strategies for comprehensive intervention. The gut–liver axis comprises a sophisticated, two‐way communication system connecting the liver and the gastrointestinal tract, playing a vital role in preserving systemic health. Gut microbiota metabolites influence both local and systemic physiological processes, impacting the intestinal epithelium, gut‐vascular barrier, hepatic sinusoids, and systemic circulation. In experimental models of MASH, microbial imbalances within the gut impair the integrity of the intestinal barrier and elevate levels of detrimental metabolites, thereby hastening the advancement of liver pathology. Given their recognized health‐promoting effects, probiotics have emerged as a promising therapeutic option for managing hepatic disorders.

Certain bacterial species, including *F. duncaniae*, have demonstrated liver‐protective properties by strengthening intestinal barrier integrity and modulating lipid metabolic processes. However, anaerobic probiotics like *F. duncaniae* are vulnerable to oxidative stress in the gastrointestinal tract, limiting their therapeutic potential. As a result, targeted delivery of probiotics remains a significant challenge. In recent years, the development of nanoscale‐based drug delivery platforms has attracted considerable attention, owing to their potential to reduce adverse effects, improve bioavailability, and enable controlled or prolonged release of therapeutic agents. Several innovative nanoplatforms have been developed, such as tungsten‐coated zinc nanoparticles for inflammatory bowel disease therapy and lipid nanoparticles (LNPs) for remodeling the MASH microenvironment. Gold nanoparticles (AuNPs) are particularly notable due to their surface reactivity, low toxicity, and bioactive properties. Chiral gold nanoparticles (c‐AuNPs) represent a novel class of nanozymes, which can mimic intracellular enzyme systems that combat oxidative stress, making them particularly effective in gastrointestinal environments.

This research conducted a comparative evaluation of the intestinal microbial communities in individuals diagnosed with MASH versus healthy subjects, identifying pronounced dysbiosis among the MASH group. Notably, there was a substantial reduction in the abundance of *Faecalibacterium*, a commensal bacterium widely recognized for its anti‐inflammatory properties. Among members of this genus, *Faecalibacterium duncaniae (F. duncaniae)* represents a well‐characterized commensal strain with prominent anti‐inflammatory bioactivity. Building upon this observation, we developed an innovative therapeutic platform: a chiral gold nanozyme‐modified probiotic system designed to modulate the gut–liver axis and reverse MASH progression. Our results demonstrate that AuNPs exhibit enzyme‐mimetic properties, including superoxide dismutase (SOD)‐like and catalase (CAT)‐like activities. These properties enable AuNPs to efficiently scavenge reactive oxygen species (ROS), thereby mitigating oxidative stress and associated inflammation. Supplementation with *F. duncaniae* in murine models improved gut microbiota composition and intestinal barrier function. Notably, the right‐handed nanozyme (D‐Au) nanoparticles protected *F. duncaniae* against oxidative stress and facilitated its intestinal colonization. This intervention ameliorated MASH, accompanied by reshaped gut microbiota and enhanced anti‐inflammatory responses. In MASH mouse models, the F@D‐Au system exerted prominent hepatoprotective effects, including ROS scavenging, reduced oxidative damage, and suppressed expression of inflammatory cytokines, alongside reversed hepatic pathological changes. Gut microbiome sequencing revealed that F@D‐Au intervention significantly reduced the *Firmicutes*/ *Bacteroidota* ratio to a level comparable to healthy controls. Mechanistic analyses further indicated that the protective effects of F@D‐Au were linked to the reprogramming of tryptophan and linoleic acid metabolic pathways. Within the tryptophan pathway, F@D‐Au treatment increased kynurenic acid and melatonin, which correlated with the alleviation of MASH lesions and the improvement of MASH‐associated cognitive dysfunction. In the linoleic acid pathway, F@D‐Au intervention elevated phosphatidylcholine and linoleic acid, which are associated with improved hepatic metabolic phenotypes.

In summary, our human microbiota analysis revealed marked gut dysbiosis in MASH patients, accompanied by decreased abundance of the *Faecalibacterium* genus. We selected *F. duncaniae*, a representative strain of this genus, to construct and screen chiral nanozyme‐probiotic hybrids, identifying the D‐Au form as uniquely potent. The resulting F@D‐Au platform demonstrated striking efficacy in murine MASH, reversing histopathology and restoring metabolic health. Beyond the liver, it ameliorated cognitive deficits; this effect was associated with the restoration of specific neuroactive metabolites. This study introduces a platform technology that merges chiral nanomedicine with synthetic microbiology, offering a powerful and versatile strategy to target complex, multi‐system diseases through the gut microbiome. The significance of this investigation lies both in the development of an innovative nano‐biological therapeutic and, crucially, in underscoring the pivotal influence of gut microbiota modulation as a central strategy for treating metabolic liver diseases, providing a technological paradigm for the development of next‐generation “smart probiotics”.

## 2 Results

### 2.1 Gut Microbiome Characterization in MASH and Healthy Individuals

To identify potential probiotics for MASH therapy, we enrolled 16 participants (7 healthy adults and 9 MASH patients). Baseline clinical characteristics were compared between healthy controls and MASH patients (Supplementary Table 1). No significant inter‐group differences were observed in age, sex distribution, and height (*p* > 0.05). Compared with healthy controls, MASH patients exhibited significantly higher body weight, body mass index(BMI), serum Alanine aminotransferase (ALT), aspartate aminotransferase (AST), triglycerides, and total cholesterol. For hepatic histological assessment, all MASH patients underwent liver biopsy, and hepatic steatosis, lobular inflammation, hepatocellular ballooning, as well as fibrosis stage were evaluated according to the NAFLD Activity Score (NAS). Liver biopsy was not performed in healthy controls; the status of absent hepatic steatosis, liver injury, and fibrosis (NAS  =  0, fibrosis stage F0) was determined based on liver biochemistry and abdominal ultrasound findings. The distributions of liver fibrosis stage, steatosis grade, lobular inflammation grade, hepatocellular ballooning grade, and total NAS score were significantly different between the two groups (all *p* < 0.001). None of the participants in either group had a medication history. High‐throughput sequencing of 16S rDNA was employed to characterize the bacterial communities present in fecal samples. Results demonstrated that compared with healthy controls, MASH patients showed significant alterations in alpha‐diversity indices (Chao1, Simpson, and Shannon) calculated at the amplicon sequence variant (ASV) level (all *p* < 0.05), indicating obvious disturbance of intestinal microbial richness and diversity in MASH subjects (Figure 1A). Using principal coordinates analysis (PCoA), we observed an obvious distinction in the clustering patterns of gut microbiota from individuals diagnosed with MASH compared to those from healthy control subjects. PERMANOVA analysis revealed significant dissimilarity between‐group dissimilarity (*p * =  0.003, *R* =  0.145). This finding indicates that the microbial community structures differ substantially between the two groups (Figure 1B). LEfSe analysis identified significant differences in microbial composition at the genus level, with *Faecalibacterium* abundance being markedly reduced in MASH patients (Figure 1C,D). Analysis using a heatmap provided additional confirmation that the levels of *Faecalibacterium* were notably lower in individuals suffering from MASH. This supports earlier findings regarding the decreased abundance of this bacterium in the patient group (Figure 1E). Quantitative assessment revealed that healthy individuals exhibited a markedly greater relative abundance of *Faecalibacterium* in comparison with those diagnosed with MASH (Figure 1F), aligning with earlier research outcomes [10, 11, 12]. *Faecalibacterium* abundance was correlated with MASH status in this small cohort, and lower levels were observed in MASH subjects. Notably, higher BMI in MASH patients may act as a confounding factor for this microbial alteration. *F. duncaniae* is the most abundant and best‐characterized representative species within this genus [13], is significantly less abundant in feces from patients with MASH, and its abundance is negatively correlated with hepatic steatosis severity and fibrosis stage [13, 14]. Previous studies have demonstrated that *F. duncaniae* can significantly reduce plasma lipopolysaccharide (LPS) levels [12] – an established marker of impaired intestinal barrier function [15]. Multiple studies have shown that *F. duncaniae* supplementation improves cognitive impairment in mouse models [16, 17]. As one of the major butyrate producers with well‐documented anti‐inflammatory properties, *F. duncaniae* has been shown to protect the digestive system against enteric pathogens [18, 19]. Based on the above lines of evidence, *F. duncaniae* was selected as a candidate probiotic strain for subsequent mechanistic investigations in MASH intervention studies.

**FIGURE 1:**
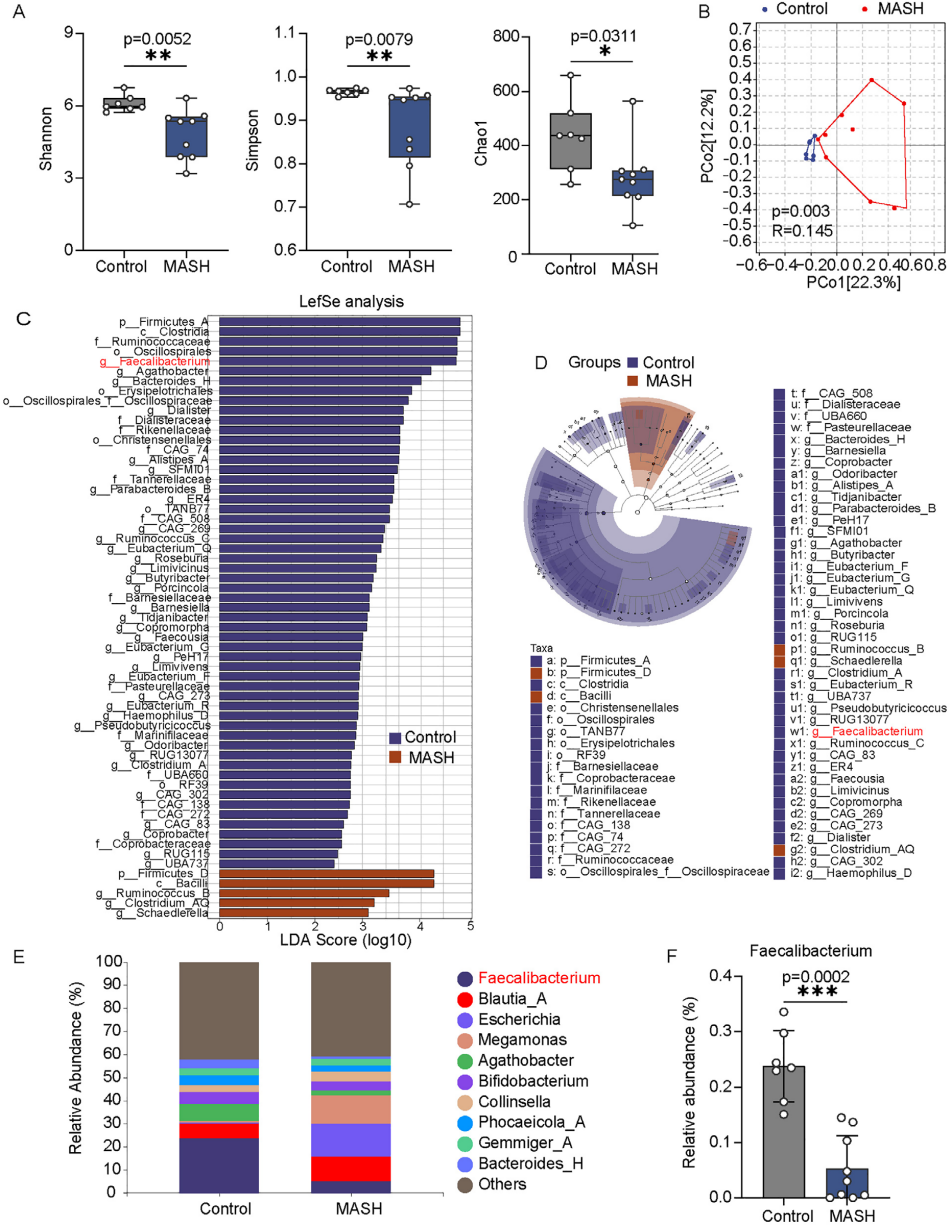
Screening of probiotics using 16S rDNA sequencing. Control (*n* = 7), MASH (*n* = 9). (A) Assessment of fecal microbiome alpha diversity among groups was performed at the amplicon sequence variant (ASV) level, utilizing the Chao1, Simpson, and Shannon indices. (B) PCoA illustrating beta diversity variations of the gut microbiota across different experimental cohorts. Group differences were assessed by PERMANOVA. (C) Identification of taxa with significant differences between Control and MASH groups, as determined by LEfSe analysis using LDA scores, where a logarithmic cutoff of 2.0 was applied. (D) Cladogram produced by LEfSe, visualizing compositional differences in gut bacterial communities among the groups. (E) Taxonomic profiling displaying the genus‐level composition and relative abundance of gut microbiota in both Control and MASH groups. (F) Quantification of the genus Faecalibacterium, depicting its relative abundance in the Control group compared to the MASH group. Comparisons between the two groups were performed using the Mann‐Whitney U test (A, F). Data are presented as mean ± standard deviation. ns, not significant (*p* > 0.05), ^*^ *p* < 0.05, ^**^ *p* < 0.01, ^***^ *p* < 0.001, ^****^ *p* < 0.0001.

### 2.2 Design and Characterization of Engineered Chiral Probiotics

We synthesized L‐A and D‐Au by slightly modifying our previously established method. (Figure 2A) [20]. Transmission electron microscopy (TEM) was utilized to characterize the morphology of the chiral gold nanozymes (Figure 2B). TEM analysis demonstrated that these nanozymes are uniform, monodisperse, and exhibit a spherical shape, with their diameters ranging between 2 and 5 nm. To determine the mean particle size, measurements were taken from 40 individual nanoparticles for both L‐Au and D‐Au. The calculated average diameter for the two types was 3.4 nm, indicating high uniformity across the samples (Figure 2C,D). Subsequently, we verified the chiral properties of the nanomaterials using circular dichroism (CD) spectroscopy. The results indicated a negative peak at 220 nm for the CD spectrum of L‐Au, whereas D‐Au displayed a positive CD spectrum at the same wavelength (Figure 2E), demonstrating the chiral nature of these gold nanoparticles. Moreover, the Ultraviolet–visible (UV–vis) absorption spectra of L‐Au and D‐Au overlapped (Figure 2F). The zeta potential values of synthesized L‐Au and D‐Au were almost identical (Figure S1). Overall, the synthesized gold nanoparticles exhibit symmetrical CD spectra, nearly identical particle sizes, UV–vis absorption, and surface charges, indicating that surface chirality is the only distinguishing feature between L‐Au and D‐Au. We then cultured pre‐screened *F. duncaniae*, which appeared rod‐shaped and approximately 1500–2000 nm long under TEM (Figure 2G), providing ample sites for gold nanozyme attachment. Since both chiral gold nanozymes and *F. duncaniae* possess negatively charged surface properties, strong electrostatic repulsion dominates their interaction and prevents direct spontaneous binding between bare nanozymes and bacterial cells. To achieve stable surface modification, chiral gold nanozymes were first functionalized with amino‐terminated polyethylene glycol. After surface amination, the modified nanozymes were covalently anchored onto the surface of *F. duncaniae* through an amide coupling reaction. Collectively, this fabrication strategy relies on broad‐spectrum, artificially mediated chemical cross‐linking, rather than biological specific recognition such as natural receptor‐ligand binding (Figure 2A). In addition, the physical interaction between unmodified free L‐Au/D‐Au nanoparticles and bacteria is predominantly governed by non‐specific electrostatic forces and hydrophobic effects. Although the intrinsic surface chirality of gold nanoparticles endows them with enantiomer‐dependent differential binding affinity toward bacterial surfaces, such chiral selective adhesion does not constitute strict, biologically specific recognition. TEM confirmed the successful attachment of L‐Au and D‐Au to the probiotics (Figure 2G). Further TEM elemental mapping and energy‐dispersive spectroscopy (EDS) analysis confirmed the abundance of Au on the engineered bacteria surface (Figure 2G–I), indicating that L‐Au and D‐Au were successfully anchored to the bacterial surface. We successfully constructed the probiotic‐artificial enzyme systems F@L‐Au and F@D‐Au.

**FIGURE 2:**
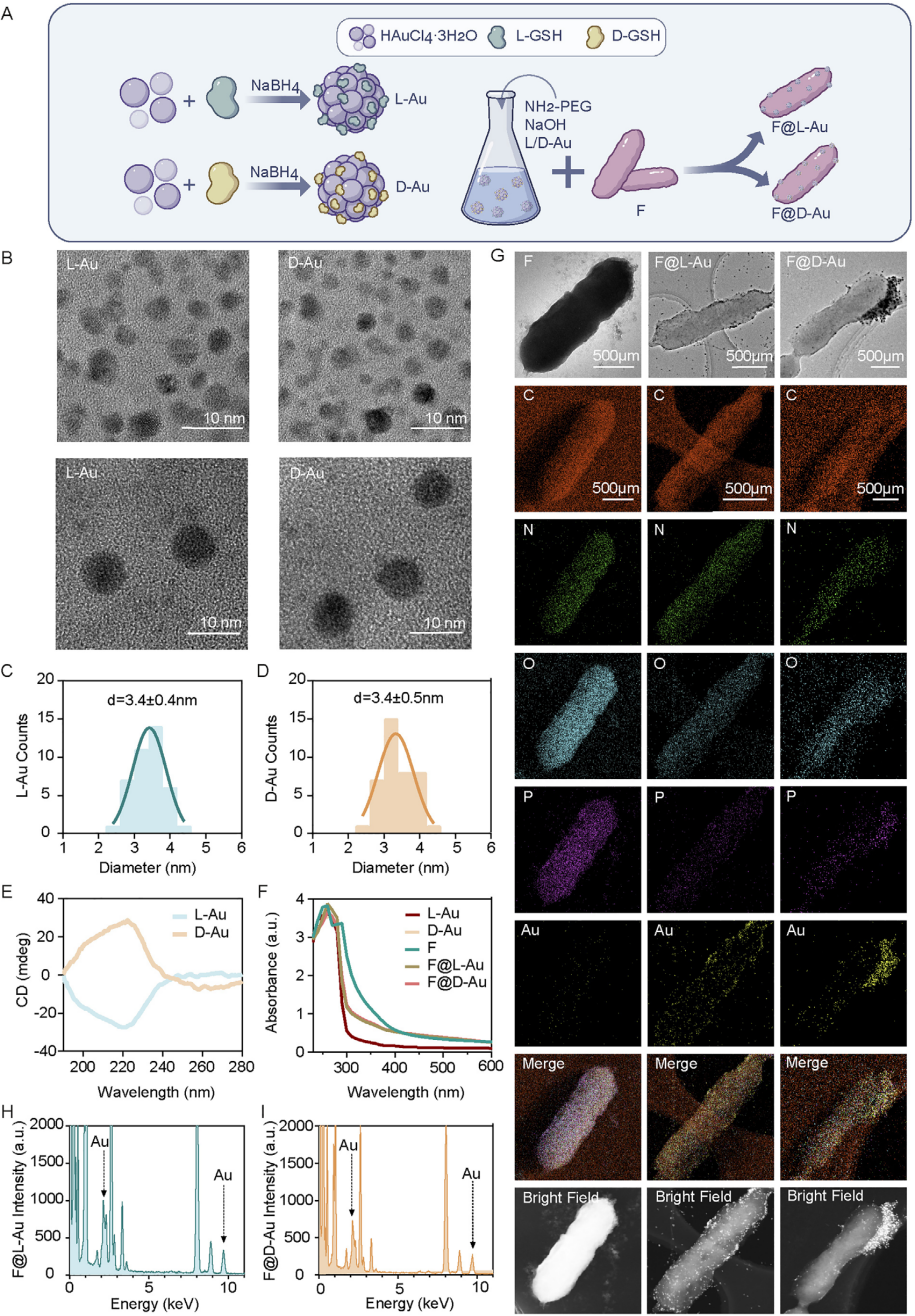
Synthesis and Structural Characterization of L‐Au, D‐Au, F (*F. duncaniae*), F@L‐Au, and F@D‐Au. (A) Adobe Illustrator was used to create a schematic diagram depicting the synthesis process for L‐Au and D‐Au nanoparticles, as well as their subsequent conjugation with F. (B) Representative TEM images display the morphology of both L‐Au and D‐Au nanoparticles; the scale bar represents 10 nm. (C) A histogram illustrating the size distribution of L‐Au nanoparticles is shown, based on measurements from 40 individual particles. (D) Size distribution histogram of D‐Au (*n* = 40 nanoparticles). (E) CD spectra were recorded for both L‐Au and D‐Au nanoparticles. (F) UV–vis absorption spectra were obtained for L‐Au, D‐Au, F, as well as for the conjugated forms F@L‐Au and F@D‐Au. (G) TEM images and corresponding elemental mapping of F@L‐Au and F@D‐Au. (H) EDS spectrum of F@L‐Au. (I) EDS spectrum of F@D‐Au.

### 2.3 Determination of Chiral Nanozyme Activity and Antioxidative Stress Performance

Extensive research has demonstrated that the performance of nanomaterials is intricately linked to factors such as their chirality. Chiral inorganic nanomaterials possess the capability to mimic enzymatic functions, acting as nanozymes. Unlike natural enzymes, nanozymes offer advantages such as enhanced reusability, tunable catalytic activity, and reduced synthesis costs, thereby addressing multiple inherent limitations of their biological counterparts [21, 22]. As a major antioxidant within the body's defense mechanisms, glutathione is essential for safeguarding tissues against oxidative injury caused by ROS [23, 24, 25]. To verify the anti‐ROS capability of our designed chiral L‐glutathione‐ and D‐glutathione‐capped gold nanoparticles, we utilized SOD and CAT detection kits to measure the SOD‐ and CAT‐mimicking activities of chiral gold nanoparticles at different concentrations. CAT and SOD are crucial ROS‐scavenging enzymes that mitigate oxidative injury by degrading peroxides in biological systems. Our assays confirmed that both chiral gold nanoparticles exhibit ROS‐scavenging capacity, and the observed ROS‐elimination effect rises with increasing nanoparticle concentration. Of note, enantiomer‐dependent antioxidant performance was observed, where D‐Au displayed relatively stronger ROS‐scavenging activity than L‐Au (Figure S2).

### 2.4 Protective Effects of L‐Au and D‐Au Against LPS‐Induced Cellular Component Damage

In vitro cytotoxicity analysis of L‐Au and D‐Au was conducted using intestinal epithelial cells (HT29 cells). After 24‐h co‐incubation with various concentrations of L‐Au and D‐Au, the results indicated minimal cytotoxicity, with cell viability exceeding 80% (Figure 3A). Effective intracellular delivery of nanomedicine is vital for therapeutic efficacy. We co‐incubated Cy3‐labeled chiral gold nanoparticles with intestinal epithelial cells, and confocal laser scanning microscopy (CLSM) revealed that the chiral gold nanozymes efficiently entered the cells, with cellular uptake increasing over time. This suggests that the nanoparticles can be efficiently internalized by cells, with no significant difference in the endocytic efficiency between L‐Au and D‐Au (Figure 3B).

**FIGURE 3:**
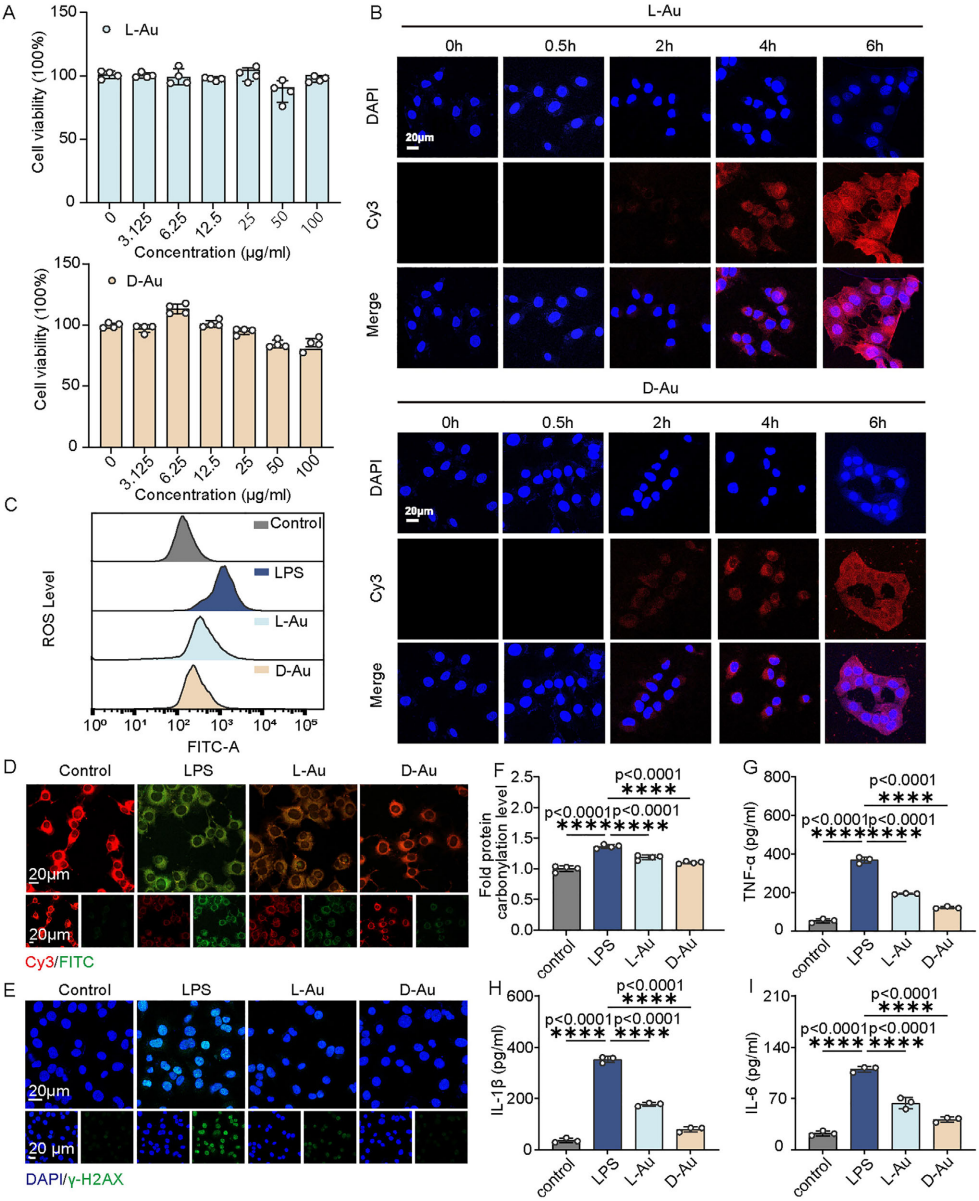
Protective Effects of L‐Au and D‐Au Against LPS‐Induced Cellular Component Damage. (A) Toxicity assessment following co‐incubation of intestinal epithelial cells with varying concentrations of L‐Au. Cytotoxicity was measured using the CCK‐8 assay (*n* = 4). Toxicity assessment following co‐incubation of intestinal epithelial cells with varying concentrations of D‐Au. Cytotoxicity was measured using the CCK‐8 assay (*n* = 4). (B) CLSM images of intestinal epithelial cells co‐incubated with L‐Au and D‐Au at 0, 0.5, 2, 4, and 6 h. Representative images from three replicates in each group are shown. (C) The ability of various materials to scavenge ROS in intestinal epithelial cells exposed to LPS‐induced oxidative stress was evaluated. This assessment was performed using flow cytometry, with DCFH‐DA serving as the fluorescent probe for ROS detection. The concentration of LPS stimulation was 1µg/mL for 12 h, and the treatment concentrations of L‐Au and D‐Au were 100µg/mL. Representative images from three replicates in each group are shown. (D) CLSM images of lipid peroxidation in intestinal epithelial cells stained with C11‐BODIPY581/591 following different treatments. Representative images from three replicates in each group are shown. (E) CLSM immunofluorescence images illustrating γ‐H2AX foci, which indicate DNA damage in intestinal epithelial cells subjected to different treatment conditions. Three replicates from each group are displayed. (F) Levels of protein carbonylation in intestinal epithelial cells following different treatments (*n* = 4). (G, H, I) Concentrations of pro‐inflammatory cytokines TNF‐α, IL‐1β, and IL‐6 were measured in the supernatants of cells after exposure to various treatment conditions (*n* = 3). Comparisons among multiple groups were conducted using one‐way analysis of variance (one‐way ANOVA), followed by Tukey's multiple‐comparisons post‐hoc test (F–I). Data are presented as mean ± standard deviation. ns, not significant (*p* > 0.05), ^*^ *p* < 0.05, ^**^ *p* < 0.01, ^***^ *p* < 0.001, ^****^ *p* < 0.0001.

Exposure to lipopolysaccharide (LPS) triggers oxidative stress, leading to an overproduction of ROS. The accumulation of these ROS disrupts normal cellular functions, which may ultimately result in both cell death and pronounced inflammatory responses. LPS‐induced ROS generation was utilized to simulate the intestinal oxidative stress state of MASH in vitro. Flow cytometric assessment demonstrated that intracellular ROS levels were markedly elevated in HT29 cells upon exposure to LPS. However, administration of either L‐Au or D‐Au significantly suppressed ROS generation, as indicated by a shift toward lower fluorescence intensity in the corresponding histograms (Figure 3C). Further observation of LPS‐induced cellular ROS damage through fluorescence microscopy and quantification of mean fluorescence intensity confirmed the potent antioxidant capacity of D‐Au (Figure S3A,B). To further understand the protective effects of chiral gold nanozymes on cells, additional in vitro studies were conducted. Overproduction of ROS within cells induces oxidative damage to the three primary classes of bioactive macromolecules: proteins, DNA, and lipids [26, 27]. Next, we assessed the protective potential of chiral gold nanozymes against intracellular lipid peroxidation, DNA damage, and protein carbonylation induced by LPS. The in vitro ROS scavenging activity of L‐Au and D‐Au suggests that chiral gold nanozymes possess protective effects on cells. Upon incubation with C11‐BODIPY581/591, cells exposed to LPS exhibited reduced red fluorescence alongside an enhancement in green fluorescence intensity (Figure 3D). In the D‐Au treatment group, no significant change in red fluorescence was observed, indicating that D‐Au alleviates intracellular oxidative stress and prevents lipid peroxidation. ROS can cause multiple types of DNA lesions, among which double‐strand breaks (DSBs) are especially detrimental to genome stability and cell viability. To examine the ability of chiral gold nanoparticles to protect against ROS‐induced DNA damage, we focused on the phosphorylation of histone H2AX at Ser139 (γ‐H2AX), a well‐established marker of DSBs. Cells pre‐treated with D‐Au showed a marked decrease in γ‐H2AX formation, suggesting that D‐Au effectively protects DNA from oxidative damage (Figure 3E). Protein carbonylation often refers to the oxidation of amino acids in proteins by ROS, leading to alterations or inactivation of protein function. We reduced LPS‐induced intracellular protein carbonylation levels using chiral gold nanoparticles, with D‐Au showing superior effects. These findings indicate that chiral gold nanoparticles are essential in preventing the accumulation of protein carbonyl groups resulting from oxidative stress caused by reactive oxygen species (Figure 3F). Subsequently, we estimated the anti‐inflammatory capability of gold nanoparticles in vitro by evaluating cytokine expression in LPS‐stimulated intestinal epithelial cells. Analysis of inflammatory factors in the supernatants of cells subjected to various treatments demonstrated that exposure to chiral gold nanoparticles markedly decreased the concentrations of the pro‐inflammatory cytokines tumor necrosis factor‐α (TNF‐α), interleukin‐1β (IL‐1β), and interleukin‐6 (IL‐6) (Figure 3G–I). These results indicate that D‐Au significantly ameliorates LPS‐induced damage to the three major biomolecules and associated pro‐inflammatory cytokine levels, outperforming L‐Au. Based on the above findings, we speculate that chiral gold nanoparticles hold therapeutic potential for MASH treatment and may protect probiotics against ROS‐mediated damage during gastrointestinal transit.

### 2.5 Biosafety Assessment and Evaluation of Intestinal Colonization by F@L‐Au and F@D‐Au Post Oral Dosing

To ensure safe biological applications, a systematic evaluation of the in vivo toxicity of L‐Au and D‐Au was performed. Healthy C57BL/6 mice were administered varying concentrations of L‐Au and D‐Au via gavage (three times a week for two weeks). Following this, both serum biochemical indices (Figure S4A,C) and hematoxylin and eosin (H&E) staining of principal organs (Figure S4B,D) were evaluated. The analyses revealed no observable differences relative to the control group, indicating that oral administration of L‐Au and D‐Au possesses good biocompatibility. The results indicated no significant differences compared to the control group, suggesting that oral administration of L‐Au and D‐Au demonstrates favorable biocompatibility.

The stability of orally administered probiotics, such as *F. duncaniae*, can be compromised by the harsh gastrointestinal environment, including inflammation, before reaching the intestines. The initial phase of this research involved assessing the proliferation of *F. duncaniae* in modified YCFA medium, in addition to its growth performance in simulated gastric fluid (SGF) and simulated intestinal fluid (SIF). Results indicated that *F. duncaniae* exhibited slow growth in gastric fluid, moderate growth in intestinal fluid, and the fastest growth in the modified medium (Figure 4A). Further experiments investigated the growth of F, F@L‐Au, F@D‐Au, F+L‐Au, and F+D‐Au in SGF and SIF. Considering that solutions may remain in the stomach for up to 4 h, we recorded the growth of F, F@L‐Au, F@D‐Au, F+L‐Au, and F+D‐Au during a 4‐h co‐culture with SGF to evaluate the protective capabilities of L‐Au, D‐Au for *F. duncaniae* (Figure 4B). The results demonstrated improved bacterial survival for both L‐Au and D‐Au‐encapsulated bacteria compared to unencapsulated bacteria, with D‐Au encapsulation showing superior survival rates. Given that solutions can remain in the intestines for approximately 8 h, the growth of F, F@L‐Au, F@D‐Au, F+L‐Au, and F+D‐Au was recorded over this period in SIF (Figure 4C). Similar to the SGF results, encapsulation with L‐Au and D‐Au enhanced bacterial survival, with D‐Au providing the highest protection. These findings suggest that chiral gold nanozymes can act as protectors for *F. duncaniae*, shielding the encapsulated bacteria from oxidative stress in the gastrointestinal tract, thereby ensuring their safe delivery to the intestines.

**FIGURE 4:**
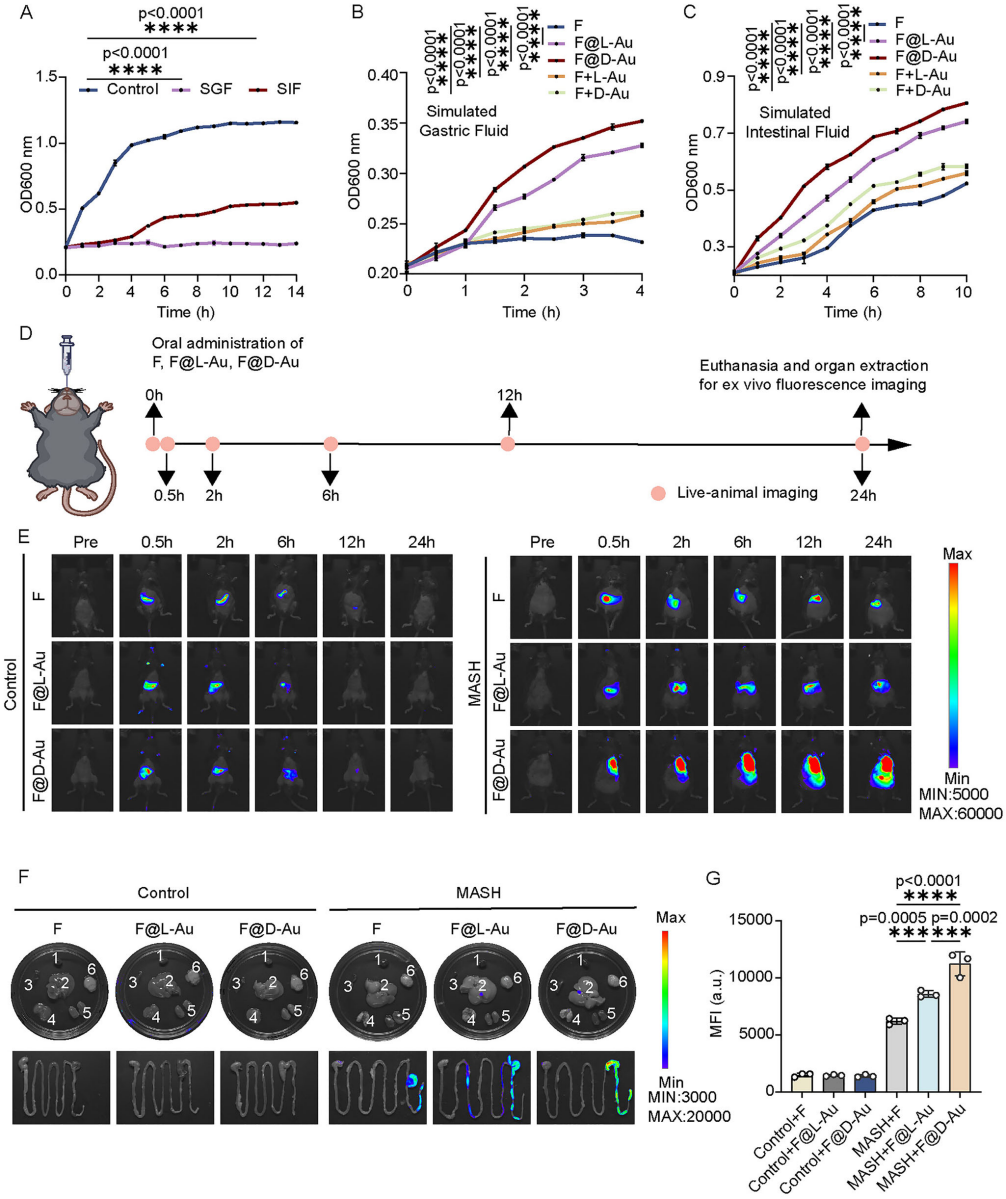
Stability and Targeting of Orally Administered F@D‐Au in the Intestine. (A) The OD values were determined using a spectrophotometer to evaluate the growth of *F. duncaniae* in modified Y‐CFA medium, SGF, and SIF. (B) In vitro resistance of F, F@L‐Au, F@D‐Au, F+L‐Au, and F+D‐Au to SGF at 37°C. The growth of engineered bacteria in SGF was tracked by measuring OD with a spectrophotometer (*n* = 3 per group). (C) In vitro resistance of F, F@L‐Au, F@D‐Au, F+L‐Au, and F+D‐Au to SIF at 37°C. The growth of engineered bacteria in SIF was assessed by measuring OD with a spectrophotometer (*n* = 3 per group). (D) Flowchart of the fluorescence imaging experiment, created using Adobe Illustrator. (E) Representative in vivo imaging of Control and MASH model mice after oral gavage with F, F@L‐Au, or F@D‐Au. (Control+F group, Control+F@L‐Au group, Control+F@D‐Au group, MASH+F group, MASH+F@L‐Au group, MASH+F@D‐Au group). (F) Representative ex vivo organ imaging (1. heart, 2. liver, 3. spleen, 4. lung, 5. kidney, 6. brain) and in vivo gastrointestinal tract images acquired by IVIS. (Control+F group, Control+F@L‐Au group, Control+F@D‐Au group, MASH+F group, MASH+F@L‐Au group, MASH+F@D‐Au group). (G) Fluorescence signal intensity in the gastrointestinal tract 24 h after oral administration (*n* = 3 per group). Comparisons were performed using two‐way analysis of variance (two‐way ANOVA) (A, B, C). Comparisons among multiple groups were conducted using one‐way analysis of variance (one‐way ANOVA), followed by Tukey's multiple‐comparisons post‐hoc test (G). Data are presented as mean ± standard deviation. ns, not significant (*p* > 0.05), ^*^ *p* < 0.05, ^**^ *p* < 0.01, ^***^ *p* < 0.001, ^****^ *p* < 0.0001.

Additionally, the in vivo retention and viability of *F. duncaniae* carried by chiral gold nanozymes were validated through fluorescence imaging in mice. After labeling F@L‐Au and F@D‐Au with IR783 fluorescent dye, they were administered to control and MASH groups of mice via gavage. In vivo imaging was used to observe the colonization of F, F@L‐Au, and F@D‐Au in different gastrointestinal regions (Figure 4D). Spectrophotometric analysis revealed no significant change in optical density (OD) before and after dye conjugation (Figure S5), indicating minimal impact of the dye on the materials. In vivo fluorescence imaging and subsequent ex vivo organ imaging were performed to evaluate the retention of orally administered F, F@L‐Au, and F@D‐Au. At 24 h post‐gavage, fluorescence signals were predominantly localized within the colon (Figure 4E,F). Both in vivo intestinal signals and ex vivo colonic fluorescence intensities exhibited obvious differences between MASH mice and healthy mice across all three treatment groups, with markedly stronger signals observed in MASH mice. In healthy mice, indigenous commensal microbiota occupy nearly all available intestinal ecological niches, generating strong colonization resistance that limits the engraftment of exogenous bacteria [28]. By contrast, gut dysbiosis in MASH mice frees substantial niches for incoming microbes, which partially supports bacterial retention [29]. Nevertheless, the inflamed MASH gut also presents a harsh microenvironment characterized by excessive ROS, which is highly toxic to the strictly anaerobic *F. duncaniae*. Notably, F@D‐Au yielded the highest fluorescence signal among formulations in MASH mice at 24 h post‐gavage (Figure 4G). This phenomenon could be explained by multi‐layered putative mechanisms. First, the superior ROS‐scavenging nanozyme activity of D‐Au provides potent antioxidative protection, acting as a “shield” for *F. duncaniae*. This protective effect may enable more bacteria to survive the oxidative stress upon arrival in the inflamed gut; sufficient survival serves as the prerequisite for subsequent colonization. Second, intestinal barrier restoration involves both epithelial tight‐junction integrity and the reconstruction of the mucus‐associated microhabitat. As *F. duncaniae* relies on the mucus layer as a metabolic niche, the inflammation‐relieving effect of F@D‐Au may help restore this mucus‐related niche [30, 31]. Rather than excluding exogenous bacteria, the rehabilitated mucus microenvironment could provide favorable physical conditions to stabilize bacterial engraftment. Collectively, niche availability, antioxidative protection, and the restoration of mucus‐associated microhabitats may jointly contribute to the elevated retention of F@D‐Au in MASH mice. Ex vivo fluorescence imaging revealed detectable hepatic signals in MASH mice following intragastric gavage of F@L‐Au and F@D‐Au (Figure 4F). To further quantify hepatic accumulation of gold‐containing components, inductively coupled plasma mass spectrometry (ICP‐MS) was performed on liver tissues harvested at 24 h after administration. Low‐but‐measurable gold elements were detected in liver samples from both the F@L‐Au and F@D‐Au groups, and no significant difference in hepatic gold concentration was observed between these two groups (Figure S6A). These results confirm that gold‐containing components from orally administered F@L‐Au and F@D‐Au can reach the liver. In the MASH mouse model, long‐term high‐fat diet disrupts intestinal epithelial barrier function and increases intestinal permeability. Although viable *F. duncaniae* mainly colonizes the gastrointestinal tract locally, gold nanozymes covalently anchored on the bacterial surface may partially detach into the gut lumen during gastrointestinal transit. These detached gold nanozymes or their fragments can cross the compromised intestinal epithelial barrier, enter the portal venous circulation, and subsequently accumulate in the liver. Accordingly, the hepatic signals detected by ex vivo fluorescence imaging and the gold quantified via ICP‐MS originate from the accumulation of detached gold nanozyme components in liver tissue, rather than translocation of intact engineered bacteria. Functionally, gold nanozyme moieties reaching the liver can directly interact with hepatic tissue to scavenge local ROS and alleviate hepatic oxidative stress, whereas the probiotic component primarily mediates immunomodulation and barrier protection locally within the gut. This spatial separation of action between the nanozyme and probiotic components highlights the synergistic therapeutic design of the engineered formulation. 24 h after gavage, quantitative real‐time polymerase chain reaction (qPCR)‐based absolute quantification of colonic mucosal *F. duncaniae* revealed that the F@D‐Au group harbored the highest bacterial loads among all intervention groups (Figure S6B). These results indicate that chiral gold nanozymes may enhance gastrointestinal persistence of *F. duncaniae* in MASH mice, which could favor its intestinal abundance and potentially contribute to favorable gut‐microbiota profiles and prolonged therapeutic benefits.

### 2.6 F@D‐Au can Ameliorate MASH Induced by CDHFD Diet in C57 Mice

We induced a MASH model in C57 mice using a choline‐deficient and high‐fat diet (CDHFD) diet for 16 weeks, establishing a treatment regimen similar to clinical MASH scenarios (Figure 5A). Treatment groups included L‐Au, D‐Au, F, F+D‐Au, F@L‐Au and F@D‐Au. In this model, F@D‐Au treatment significantly alleviated CDHFD‐induced weight gain, with a reduction of more than 10% compared to the model group (Figure 5B). We monitored fasting blood glucose levels after a 10‐h fasting period to assess blood glucose changes, revealing that post‐treatment levels with F@D‐Au were closest to those of the control group (Figure S7A), indicating effective management of MASH‐induced hyperglycemia. Subsequent insulin level measurements showed that F@D‐Au‐treated mice had insulin levels closest to those of normal mice (Figure S7B). Furthermore, glucose tolerance test (GTT) and insulin tolerance test (ITT) results showed that the F@D‐Au group exhibited improved glucose metabolism and insulin sensitivity, with values approaching the levels observed in healthy mice. However, these ameliorative trends were not statistically significant in some comparisons (Figure S7C,D). Furthermore, AUC analyses of glucose and insulin profiles showed improvements in glucose metabolism in the F@D‐Au group, with statistically significant differences observed relative to the MASH model group (Figure S7E,F). Collectively, F@D‐Au effectively ameliorated CDHFD‐induced hyperglycemia and insulin resistance. After sacrificing the mice, we collected serum and liver samples for biochemical and histopathological analysis. Images of mouse physique and liver morphology indicated reductions in body and liver size post‐treatment (Figure S8A). Liver weight, body weight, and liver‐to‐body weight ratio analysis demonstrated weight reductions in the treatment groups, with F@D‐Au achieving the most significant reduction (Figure S8B).

**FIGURE 5:**
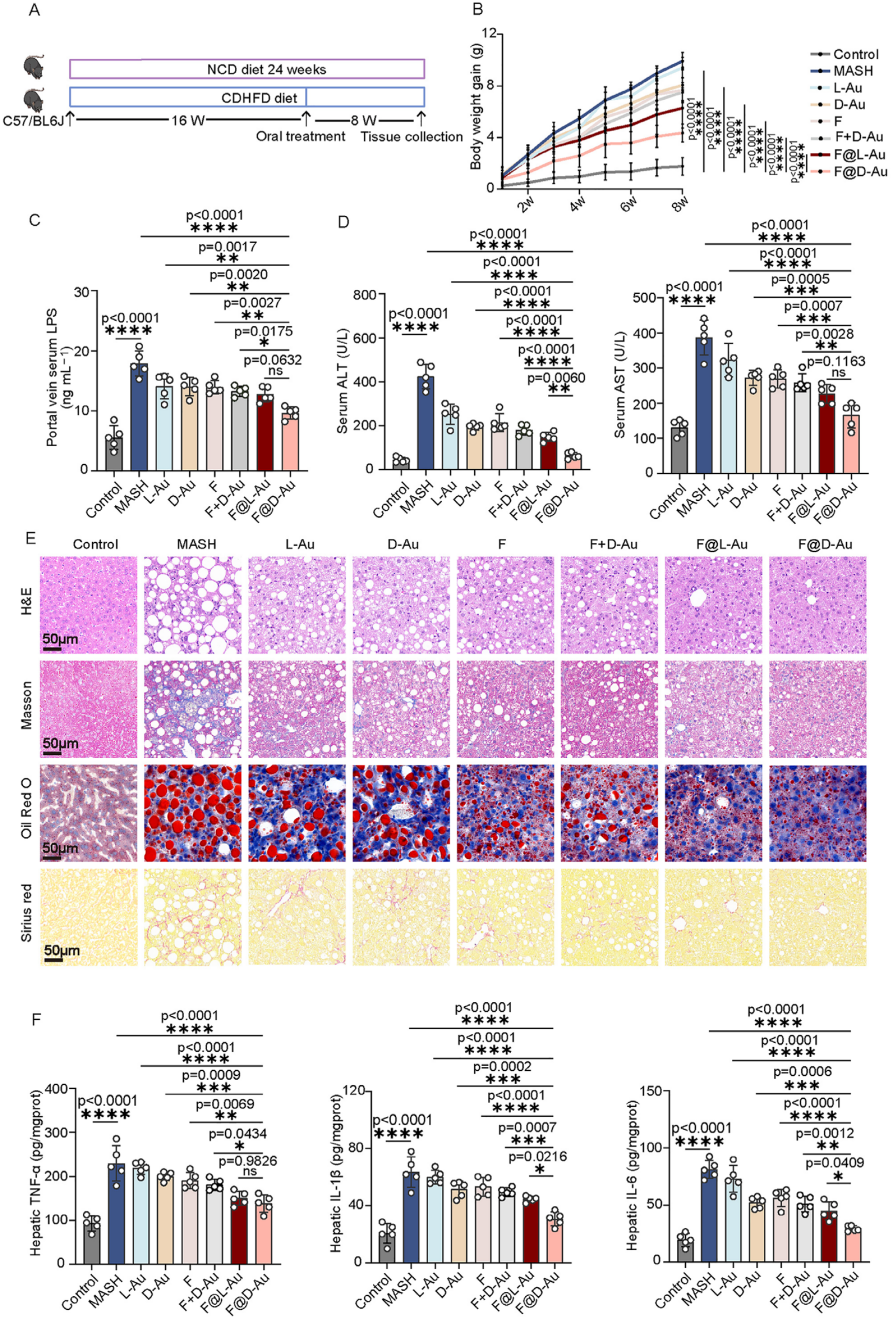
F@D‐Au Ameliorates MASH Induced by CDHFD in C57 Mice. Grouping: Control, MASH, L‐Au, D‐Au, F, F+D‐Au, F@L‐Au, F@D‐Au. (A) To establish the MASH model in C57 mice, a CDHFD was administered. Figures illustrating the study were created with Adobe Illustrator. (Treatment regimen: three times weekly). (B) Body weight curve changes under different treatments (*n* = 10). Comparisons were performed using two‐way analysis of variance (two‐way ANOVA), followed by Sidak's multiple‐comparisons test for post‐hoc pairwise comparisons. (C) Portal vein serum LPS concentrations were measured (*n* = 5). (D) Levels of serum ALT and AST were evaluated (*n* = 5). (E) Liver samples from C57 mice fed with a CDHFD underwent histological analysis using H&E, Masson's trichrome, Sirius Red, and Oil Red O staining techniques. A scale bar of 50 µm is provided. Representative images from three independent replicates of each treatment group are presented. (F) Quantification of TNF‐α, IL‐1 β, and IL‐6 in hepatic tissues (*n* = 5). Comparisons among multiple groups were performed using one‐way analysis of variance (one‐way ANOVA), followed by Tukey's multiple‐comparisons post‐hoc test (C, D, F). Data are presented as mean ± standard deviation. ns, not significant (*p* > 0.05), ^*^ *p* < 0.05, ^**^ *p* < 0.01, ^***^ *p* < 0.001, ^****^ *p* < 0.0001.

Gut dysbiosis‐induced intestinal barrier dysfunction is a critical factor in MASH development. We explored whether F@D‐Au affects intestinal barrier function in the MASH context by measuring portal vein serum levels of bacterial‐derived LPS, an indicator of barrier function. CDHFD elevated LPS levels, which were reversed by all treatments, with F@D‐Au showing the most significant reversal (Figure 5C), suggesting restoration of intestinal barrier function.

Serum analysis showed improvements in ALT and AST levels in the treatment groups, with F@D‐Au showing the most substantial effect (Figure 5D). Improvements in serum triglyceride (TG), total cholesterol (TC), free fatty acid (FFA), low‐density lipoprotein (LDL), and high‐density lipoprotein (HDL) levels were observed, particularly in the F@D‐Au treatment group (Figure S9A). Histopathological examination of hepatic samples by H&E, Masson's trichrome, Oil Red O, and Sirius Red staining revealed that administration of F@D‐Au led to a marked reduction in hepatic steatosis, inflammation, hepatocyte ballooning, and liver fibrosis in the MASH model induced by CDHFD (Figure 5E). Oral administration of F@D‐Au significantly reduced the levels of pro‐inflammatory cytokines TNF‐α, IL‐1β, and IL‐6 in the liver and serum of mice (Figure 5F and Figure S9C), serum C‐reactive protein levels also decreased (Figure S9B), indicating a significant reduction in CDHFD‐induced inflammatory responses by F@D‐Au. F@D‐Au notably reduced hepatic TC, TG, and hydroxyproline levels, with these hepatic parameters closest to those of the control group among all treatment regimens (Figure S10A,B). Additionally, the reduction in hepatic malondialdehyde (MDA), thiobarbituric acid reactive substances (TBARS) levels indicated that F@D‐Au markedly alleviated CDHFD‐triggered oxidative stress, with these oxidative stress markers closest to those of the control group (Figure S10C). In summary, F@D‐Au administration markedly improves MASH and associated metabolic disturbances caused by diet in mice, exhibiting greater therapeutic effectiveness than the alternative groups evaluated.

### 2.7 F@D‐Au Significantly Improves Cognitive Function in MASH Mice

MASLD is a multisystem disorder that not only causes liver damage but may also lead to various extrahepatic complications, including diabetes, cardiovascular diseases, kidney disorders, and neurological abnormalities. In recent years, the cognitive impairment associated with MASLD has garnered increasing attention [32, 33, 34]. Therefore, we further investigated whether F@D‐Au could alleviate the cognitive dysfunction associated with MASH. The Morris water maze assay was first performed to evaluate spatial learning and memory ability (Figure 6A). The results indicated that MASH mice required a longer time to locate the platform compared to healthy mice during the training period. After 8 weeks of oral F@D‐Au administration, the escape latency was significantly shortened. Notably, F@D‐Au exhibited superior ameliorative effects compared with other treatment groups, indicating that F@D‐Au exerts a therapeutic effect on cognitive impairment in MASH mice (Figure 6B). Additionally, during the testing phase, MASH mice crossed the platform fewer times, spent more time on the platform, and had longer latency times to enter the platform compared to healthy mice. These measures were notably improved in mice after 8 weeks of F@D‐Au treatment (Figure 6C–E). Moreover, detailed evaluation of Morris water maze performance revealed a higher frequency of entries into the target quadrant and shorter escape latency in the F@D‐Au group relative to other intervention groups following eight‐week oral administration, with these parameters reaching statistical significance. In contrast, the duration spent within the target quadrant showed no statistically significant inter‐group difference (Figure S11). Following this, mice with MASH were assessed for cognitive performance and memory retention using the novel object recognition (NOR) paradigm (Figure 6F). Compared with other intervention groups, mice receiving oral F@D‐Au treatment showed higher exploration time, discrimination index, and preference index toward novel objects, with test results approaching those of normal mice (Figure 6G–I). These observations implied improved cognitive performance and memory retention upon F@D‐Au treatment in MASH mice relative to other intervention groups. Collectively, these behavioral test outcomes suggest that mice following F@D‐Au treatment exhibited more evident object‐directed exploration and better memory capabilities when compared with other intervention groups, preliminarily indicating that F@D‐Au can alleviate MASH‐induced cognitive impairment in mice to some extent.

**FIGURE 6:**
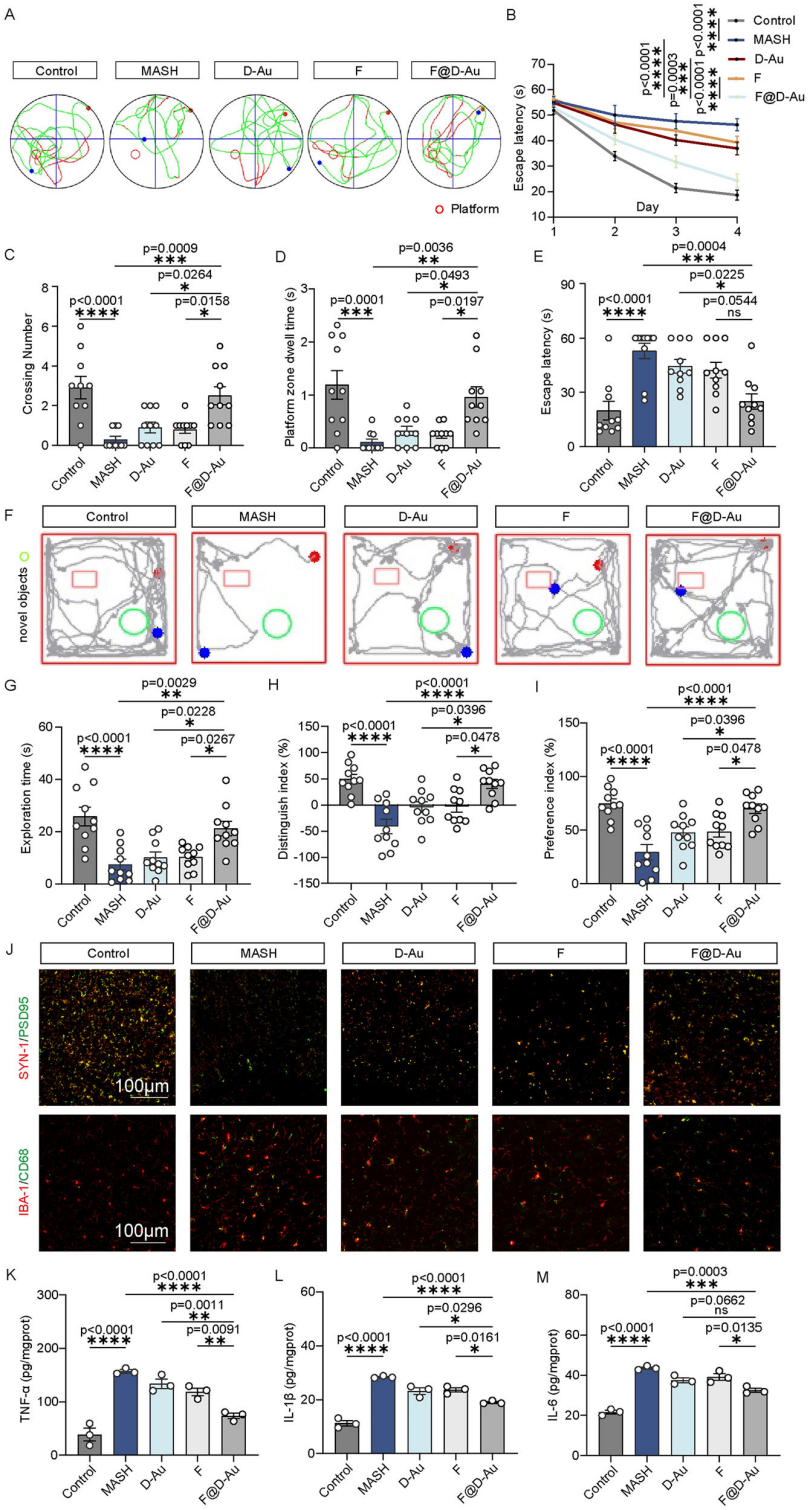
F@D‐Au Significantly Improves Cognitive Function in MASH Mice. Grouping: Control, MASH, D‐Au, F, F@D‐Au. (A) Example movement tracks obtained from the Morris water maze assessment (*n* = 10). (B) Escape latency during the acquisition phase of the Morris water maze. Comparisons among multiple groups were performed using two‐way repeated‐measures ANOVA, followed by Tukey’s multiple‐comparisons test for post‐hoc pairwise comparisons. Data are presented as mean ± SEM. (C) Crossing number during the probe trial of the Morris water maze. (D)Platform zone dwell time during the probe trial of the Morris water maze. (E) Escape latency during the probe trial of the Morris water maze. (F) Representative trajectory plots from the novel object recognition test (*n* = 10). (G) Exploration time for novel and familiar objects in the novel object recognition test. (H) Discrimination index in the novel object recognition test. (I) Preference index in the novel object recognition test. (J) Double‐label immunofluorescence for synaptic proteins SYN‐1 (red) and PSD95 (green) in hippocampal sections. Co‐localization analysis of microglial marker IBA‐1 (red) and lysosomal marker CD68 (green) in the hippocampus using immunofluorescence; scale bar corresponds to 100µm. (K, L, M) Quantification of pro‐inflammatory cytokines TNF‐α, IL‐1β, and IL‐6 in hippocampal tissue samples (*n* = 3 per group). Comparisons among multiple groups were performed using one‐way analysis of variance (one‐way ANOVA), followed by Tukey's multiple‐comparisons post‐hoc test (C, D, E, G, H, I, K, L, M). Data are presented as mean ± SEM (C, D, E, G, H, I). Data are presented as mean ± standard deviation (K, L, M). ns, not significant (P>0.05), ^*^ *p* < 0.05, ^**^ *p* < 0.01, ^***^ *p* < 0.001, ^****^ *p* < 0.0001.

Given the evident behavioral amelioration in F@D‐Au treated mice, we further explored the potential mechanism underlying the improvement of MASH‐associated cognitive deficits. Blood–brain barrier (BBB) disruption is a critical trigger of neuroinjury and cognitive decline. Therefore, BBB permeability was quantitatively evaluated by detecting Evans blue leakage and serum S100 calcium‐binding protein β (S100β) levels. The results showed that the MASH model group exhibited increased Evans blue infiltration and elevated S100β concentration, indicating severe BBB damage. Compared with all other intervention groups, the F@D‐Au group displayed the lowest Evans blue leakage and S100β concentration (Figure S12A,B). These data suggest that F@D‐Au mitigates BBB impairment under MASH conditions.

Chronic systemic inflammation has been highlighted as an important factor in the development of cognitive dysfunction in MASLD. The prevailing theory is that systemic inflammation may extend to the brain, triggering neuroinflammation and ultimately leading to cognitive impairments [33, 35]. We found that F@D‐Au treatment significantly reduced systemic inflammation in MASH mice. Therefore, we further investigated whether MASH mice exhibit brain inflammation and neuronal damage, and explored the potential effects of F@D‐Au on these conditions. Subsequently, we examined the impact of F@D‐Au on brain inflammation and neuronal health in MASH mice. First, astrocytes were labeled using glial fibrillary acidic protein (GFAP) for immunofluorescence staining of the hippocampus (Figure S13A). Results indicated higher GFAP labeling intensity and extensive astrocyte activation in the MASH group, demonstrating severe brain inflammation induced by the CDHFD diet. Compared with other intervention groups, the F@D‐Au group exhibited a more obvious reduction in GFAP labeling intensity, with staining levels close to those of the control group. Next, mature neurons were labeled with microtubule‐associated protein 2 (MAP2) for immunofluorescence staining of hippocampal neurons (Figure S13B). The immunofluorescence results revealed markedly diminished MAP2 fluorescence signals in the hippocampus of MASH mice, accompanied by fragmented neuronal morphology, suggestive of neuronal injury. Compared with other intervention groups, F@D‐Au treatment effectively increased the fluorescence intensity of hippocampal neurons, which was comparable to that of the control group. Furthermore, neurons in the F@D‐Au group displayed more intact neuronal connections without evident structural disruption. These observations indicate that F@D‐Au treatment alleviates neuroinflammatory alterations and preserves neuronal structural integrity in MASH mice. Analysis of synaptic density, based on the colocalization of the presynaptic marker synaptophysin 1 (SYN‐1) and the postsynaptic marker PSD95, revealed increased hippocampal synapse density in F@D‐Au‐treated mice compared with the MASH group, with greater improvement relative to other intervention groups (Figure 6J). Microglial activation was assessed by quantifying puncta of the lysosomal marker CD68, and the results showed reduced CD68 expression within IBA‐1‐positive microglia in the F@D‐Au group versus the MASH group, and the reduction was more evident than in other intervention groups (Figure 6J). Quantitative analysis using enzyme‐linked immunosorbent assay (ELISA) demonstrated higher concentrations of hippocampal pro‐inflammatory cytokines, including TNF‐α, IL‐1β, and IL‐6, in MASH mice. F@D‐Au treatment lowered the levels of these pro‐inflammatory cytokines in hippocampal tissue; this suppressive effect was more pronounced relative to other intervention groups (Figure 6K–M). Collectively, these observations suggest that F@D‐Au ameliorates hippocampal pathological alterations associated with CDHFD‐related cognitive impairment.

### 2.8 Mechanistic Insights into F@D‐Au‐Mediated Amelioration of MASH and Associated Cognitive Impairment

The gut microbiome has emerged as a crucial factor in gut–liver axis interactions. To evaluate how oral administration of F@D‐Au influences the structure and diversity of gut microbial communities, we utilized high‐throughput sequencing of the 16S rRNA gene from murine fecal samples. Analysis of alpha diversity metrics, including Chao1, Shannon, and Simpson indices, indicated that there were no statistically significant differences in microbial diversity between the F@D‐Au treated group and the controls. Nevertheless, notable differences were observed between the treatment and MASH groups (Figure S14A–C). This suggests that F@D‐Au treatment can shift the gut microbiota diversity of MASH mice toward that of healthy mice. Analysis of beta diversity using principal component analysis (PCA) demonstrated clear distinctions in gut microbial community structure among the MASH, control, and F@D‐Au‐treated mice. Notably, the treatment and control groups displayed gut microbiota profiles that were more closely aligned with each other compared to the MASH group (Figure 7A). When analyzed at the phylum level, mice receiving F@D‐Au treatment showed an increased abundance of *Bacteroidota* and a decreased proportion of *Firmicutes* relative to those in the MASH group (Figure 7B,C). The gut microbiota, consisting of over 1000 bacterial species, is primarily composed of the phyla *Firmicutes* and *Bacteroidota*, which together account for 90% of the microbial community. Diseases can lead to alterations in the relative abundance of these two phyla, and the *Firmicutes/ Bacteroidota* ratio (F/B) is considered a critical factor for maintaining gut homeostasis. Previous studies have shown that obese individuals have higher *Firmicutes* abundance, whereas non‐obese individuals have higher *Bacteroidota* abundance; obesity often results in an increased F/B ratio [36, 37, 38, 39], consistent with our findings. Community heatmap analysis revealed the relative abundance of microbiota at both the phylum and genus levels, illustrating that F@D‐Au treatment in MASH mice led to a gut microbiota composition that more closely resembles that of the control group, while also showing distinct bacterial communities (Figure S15A,B). Clustering analyses at these levels consistently supported the conclusion that F@D‐Au effectively remodels the gut microbiota, offering therapeutic effects in MASH mice.

**FIGURE 7:**
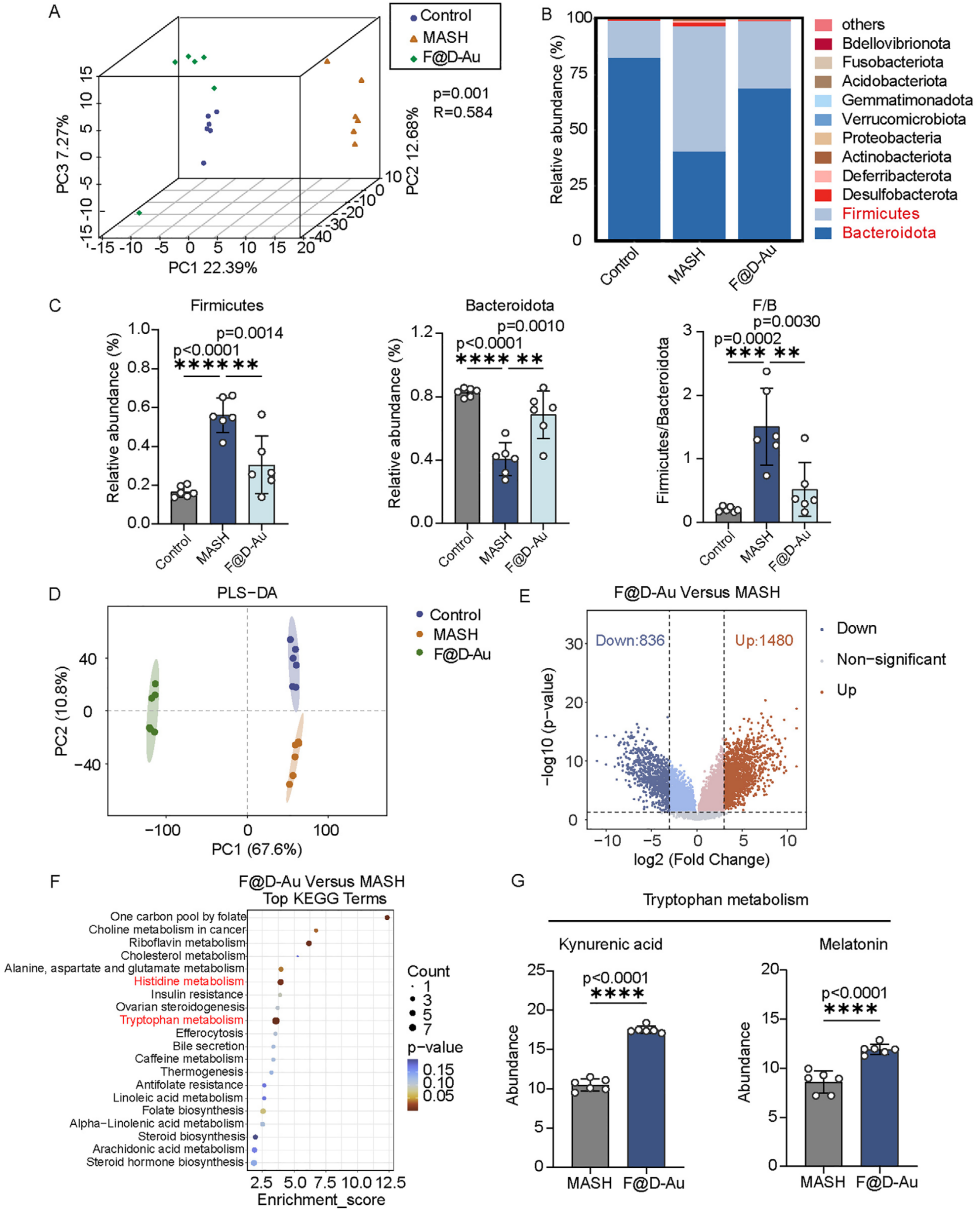
Illustration of the 16S rDNA amplicon sequencing and fecal metabolomics analysis. (A) PCA illustrating gut microbiota diversity and composition at the OTU level. Group differences were assessed by PERMANOVA. (B) The proportion of microbial communities at the phylum level was compared among the Control, MASH, and F@D‐Au cohorts. (C) The relative abundances of *Firmicutes* and *Bacteroidota* were expressed as a percentage of total microbial sequences following different interventions, with the ratio of *Firmicutes* to *Bacteroidota* (F/B) subsequently determined. (D) Partial least squares discriminant analysis (PLS‐DA) of gut‐derived metabolites was conducted, with 95% confidence intervals for each treatment group depicted by ellipses. (E) Metabolic discrepancies between the F@D‐Au and MASH groups were illustrated using a volcano plot, where upregulated metabolites unique to the experimental condition are marked in red, significantly downregulated metabolites are shown in blue, and those without notable change are indicated in gray. (F) Bubble chart of metabolic pathways significantly enriched with differential metabolites between the F@D‐Au and MASH groups. (G) Differences in major differential metabolites within the tryptophan metabolism pathway between the MASH and F@D‐Au. Comparisons among multiple groups were performed using one‐way analysis of variance (one‐way ANOVA), followed by Tukey's multiple‐comparisons post‐hoc test (C). Comparisons between the two groups were performed using the unpaired t‐test (G). Data are presented as mean ± standard deviation. ns, not significant (p>0.05), ^*^ *p* < 0.05, ^**^ *p* < 0.01, ^***^ *p* < 0.001, ^****^ *p* < 0.0001.

Therefore, we further analyzed the changes in metabolites in fecal samples from mice subjected to different treatments. Untargeted metabolomics was employed to quantify the metabolites present in mouse fecal samples. To overcome some of the inherent limitations associated with PCA, we utilized partial least squares discriminant analysis (PLS‐DA), a supervised classification approach, which allowed for a clear separation of metabolite profiles between the MASH group and both the control and F@D‐Au‐treated mice following 8 weeks of oral intervention. The treatment and control groups, in contrast to MASH, exhibited metabolomic signatures that were closely aligned to one another (Figure 7D). Analysis with a volcano plot indicated that, relative to the MASH group, F@D‐Au‐treated mice displayed an upregulation of 1480 metabolites and a downregulation of 836 metabolites (Figure 7E).

To systematically characterize metabolic reprogramming upon F@D‐Au intervention in the MASH model, pathway enrichment analysis was performed using Metabo Analyst 5.0 combined with the KEGG database. Enrichment results indicated obvious remodeling of the tryptophan metabolism pathway following F@D‐Au treatment (Figure 7F). To further profile metabolic alterations within this pathway, we detected core metabolites of tryptophan metabolism. Compared with the MASH model group, significant up‐regulation of kynurenic acid and melatonin was observed after F@D‐Au intervention (P < 0.05) (Figure 7G). Previous in vitro and animal studies have confirmed that kynurenic acid can ameliorate high‐fat‐induced metabolic abnormalities, reduce pro‐inflammatory factor expression, enhance antioxidant capacity, and alleviate hepatic inflammatory injury via GPR35 signaling [40, 41, 42]. Accumulating evidence also demonstrates that melatonin exhibits protective potential against cognitive impairment in multiple animal models [43, 44]. Our untargeted metabolomic data showed that elevated kynurenic acid and melatonin were accompanied by the amelioration of MASH lesions and the improvement of secondary cognitive phenotypes. Tryptophan metabolic reprogramming therefore represents an important metabolic feature associated with the protective effects of F@D‐Au.

KEGG enrichment also indicated alterations in histidine metabolism, another gut‐microbiota‐relevant pathway closely linked to MASH progression [45]. Clinical evidence has revealed that disturbed microbial histidine catabolism contributes to hepatic steatosis and inflammatory responses in MASH. Microbe‐derived histidine‐related metabolites can aggravate intestinal barrier disruption and amplify systemic inflammatory stress, further aggravating liver injury. In our study, changes in histidine‐metabolic signatures were observed upon F@D‐Au treatment, which may represent another relevant metabolic feature accompanying MASH improvement.

Subsequently, we conducted untargeted metabolomics analysis on mouse serum metabolites, using the same methodology as for mouse fecal metabolites. PLS‐DA revealed that the metabolic profiles of the MASH group differed markedly from those observed in both the control and treatment groups (Figure S16A). The volcano plot indicated that, compared to MASH mice, F@D‐Au‐treated mice exhibited 435 upregulated and 392 downregulated metabolites (Figure S16B). The heatmap demonstrated that serum metabolites in MASH mice were significantly altered after 8 weeks of oral F@D‐Au treatment, consistent with changes in gut microbiota and intestinal metabolites (Figure S16C). KEGG pathway enrichment analysis and screening results showed significant alterations in the linoleic acid metabolic pathway (Figure S16D). To gain deeper insight into individual metabolites, we quantified the levels of representative compounds involved in the linoleic acid metabolism pathway. The results indicated that the levels of phosphatidylcholine (Figure S16E) and linoleic acid (Figure S16F) were significantly elevated in this pathway. Phosphatidylcholine (PC) plays a vital role in preserving hepatic function. Evidence suggests that diminished hepatic phospholipid content may be a contributing factor in the development of MASLD, which has the potential to advance to MASH [46]. Administration of phosphatidylcholine has been shown to significantly attenuate the progression of metabolic dysfunction‐associated hepatic steatosis [47]. Additionally, research has suggested that reduced levels of phosphatidylcholine can be used to diagnose MASH [48]. The accumulation of cholesterol plays a pivotal role in driving the transition from hepatic steatosis to MASH [49, 50, 51]. Elevated levels of cholesterol markedly exacerbate cytotoxicity and enhance both pro‐inflammatory and pro‐fibrotic responses within hepatocytes, immune cells, and hepatic stellate cells (HSCs) [52]. Attenuation of MASH progression can be achieved by suppressing cholesterol synthesis and intestinal uptake, or by promoting cholesterol efflux from hepatic tissues. Linoleic acid has the capacity to regulate serum cholesterol levels, with an increasing content of linoleic acid correlating with a downward trend in serum cholesterol levels [53, 54, 55]. Lower linoleic acid levels are associated with cognitive impairment, and metabolic disorders contribute to cognitive dysfunction following traumatic brain injury. Moreover, serum linoleic acid has a regulatory effect on blood glucose, with its levels being inversely correlated with fasting glucose.

F@D‐Au administration is accompanied by shifts in gut microbiome composition, characterized by increased relative abundance of *Bacteroidota* and decreased *Firmicutes*, with the microbial profile showing greater similarity to that of healthy controls. The therapeutic benefits against MASH are correlated with alterations in fecal tryptophan metabolism, alongside elevated kynurenic acid. Disturbances in histidine‐associated metabolic profiles are also correlated with the therapeutic outcome. Meanwhile, increased melatonin is observed, which is associated with the improvement of MASH‐triggered cognitive impairments. Moreover, the protective outcomes are associated with changes in serum linoleic acid metabolism, including up‐regulated phosphatidylcholine. Higher linoleic acid levels are also correlated with reduced cholesterol, modulated blood glucose, and alleviated MASH‐related cognitive dysfunction. Collectively, these findings suggest that the mitigation of MASH by F@D‐Au is associated with reshaped gut microbiota and corresponding metabolic signatures.

### 2.9 F@D‐Au Ameliorates Diet‐Induced MASH and Concurrent Cognitive Impairment in SD Rats

We established a MASH model in Sprague Dawley (SD) rats by feeding them a CDHFD for 16 weeks, followed by an 8‐week oral therapeutic intervention to mimic clinical MASH therapeutic protocols (Figure 8A). After treatment, liver ultrasonography was performed on the SD rats. We assessed liver echogenicity by observing the hepatorenal interface and evaluated hepatic attenuation via the maximum cross‐sectional area of the right hepatic lobe. The results demonstrated that F@D‐Au treatment significantly improved fatty liver conditions(Figure S17). Following treatment, the rats were euthanized, and liver tissues were harvested for pathological examination. Histological changes induced by F@D‐Au treatment were assessed using a variety of staining techniques, including H&E, Masson's trichrome, Sirius Red, as well as Oil Red O staining. The findings demonstrated that F@D‐Au treatment markedly alleviated hepatic steatosis, necroinflammation, ballooning degeneration, and fibrosis in the CDHFD‐induced MASH model (Figure S18), consistent with the therapeutic effects observed in the C57 mouse model.

**FIGURE 8:**
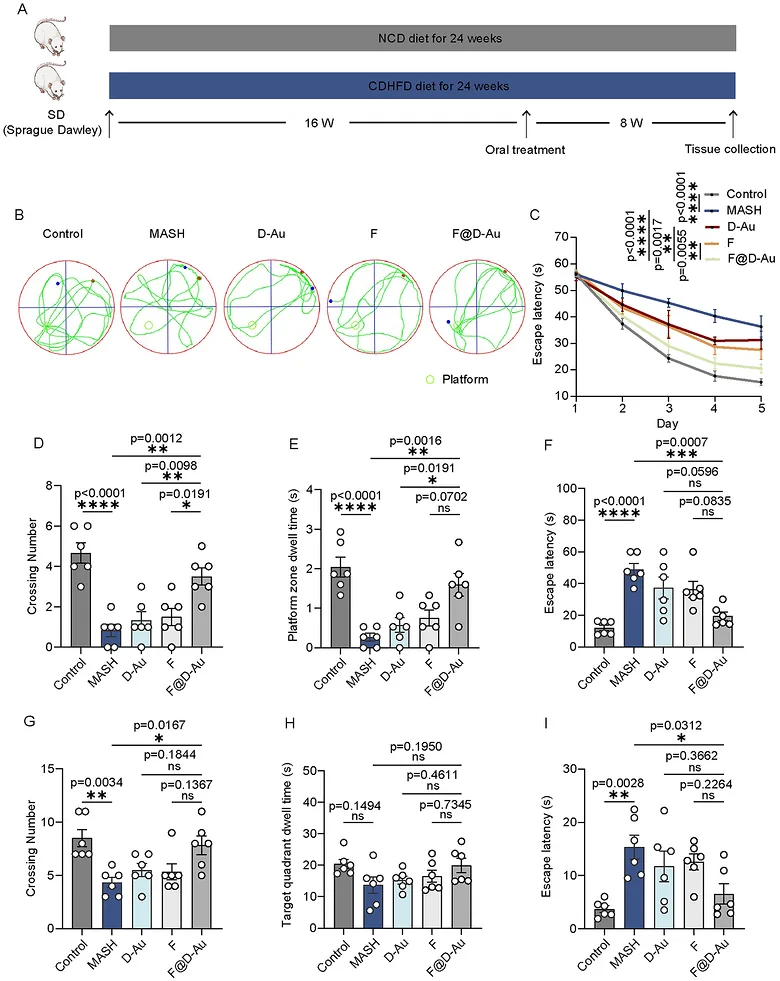
Oral administration of F@D‐Au for 8 weeks significantly improves MASH and associated cognitive impairment in SD rats. Grouping: Control (*n* = 6), MASH (*n* = 6), D‐Au (*n* = 6), F (*n* = 6), F@D‐Au (*n* = 6). (A) Schematic diagram of the experimental protocol for oral treatment in CDHFD – induced MASH in SD rats. (Treatment regimen: three times per week). Created using Adobe Illustrator. (B) Example movement tracks obtained from the Morris water maze assessment. (C) Escape latency during the acquisition phase of the Morris water maze.Comparisons among multiple groups were performed using two‐way repeated‐measures ANOVA, followed by Tukey’s multiple‐comparisons test for post‐hoc pairwise comparisons. Data are presented as mean ± SEM. (D) Crossing number during the probe trial of the Morris water maze. (E)Platform zone dwell time during the probe trial of the Morris water maze. (F) Escape latency during the probe trial of the Morris water maze. (G) Crossing number of the target quadrant during the Morris water maze test. (H) Dwell time in the target quadrant during the Morris water maze test. (I) Escape latency to the target quadrant during the Morris water maze test. Comparisons among multiple groups were performed using one‐way analysis of variance (one‐way ANOVA), followed by Tukey's multiple‐comparisons post‐hoc test (D, E, F, G, H, I). Data are presented as mean ± SEM. ns, not significant (*p* > 0.05), ^*^ *p* < 0.05, ^**^ *p* < 0.01, ^***^ *p* < 0.001, ^****^ *p* < 0.0001.

Subsequently, to evaluate cognitive function in MASH rats after administration of F@D‐Au, we employed the Morris water maze test (Figure 8B). The findings indicated that during the training phase, MASH rats took significantly longer to locate the platform compared to healthy rats, whereas rats treated with F@D‐Au for 8 weeks exhibited markedly reduced platform localization time relative to other treatment groups (Figure 8C). During the testing phase, MASH rats exhibited fewer crossings over the platform, spent less time in the platform quadrant, and displayed longer latency to reach the platform compared to healthy rats. Notably, these behavioral parameters were significantly improved in the F@D‐Au treatment group after 8‐week administration when compared with other treatment groups (Figure 8D–F). Systematic analysis of the number of entries, dwell time, and entry latency in the target quadrant also confirmed modestly higher number of entries and shorter entry latency with statistical significance in the F@D‐Au‐treated group compared with other treatment groups (Figure 8G,I). In contrast, no statistically significant inter‐group difference was observed for dwell time in the target quadrant (Figure 8H). The water maze results validated that F@D‐Au improves cognitive function, providing preliminary evidence that “this material alleviates MASH‐induced cognitive impairment in rats”. In summary, the oral delivery of F@D‐Au markedly mitigated both MASH induced by diet and the related cognitive deficits observed in rats.

The experimental results demonstrated that F@D‐Au exhibited significant therapeutic effects in both C57BL/6 mouse and SD rat MASH models, indicating its broad applicability and promising potential for further clinical translation.

### 2.10 Schematic Illustration of the Design and Therapeutic Mechanism of F@D‐Au in MASH and Associated Cognitive Impairment

We constructed schematic diagrams to illustrate the fabrication of chiral Au nanozyme‐engineered *F. duncaniae* and its therapeutic mechanisms against MASH and associated cognitive impairment (Figure 9). 16S rDNA sequencing of fecal samples from healthy controls and MASH patients was performed to identify *F. duncaniae* as a key differential bacterial species. Based on this finding, L‐Au and D‐Au nanozymes were prepared with L‐GSH and D‐GSH modification, followed by covalent conjugation to *F. duncaniae* to construct F@L‐Au and F@D‐Au (Figure 9A).

**FIGURE 9:**
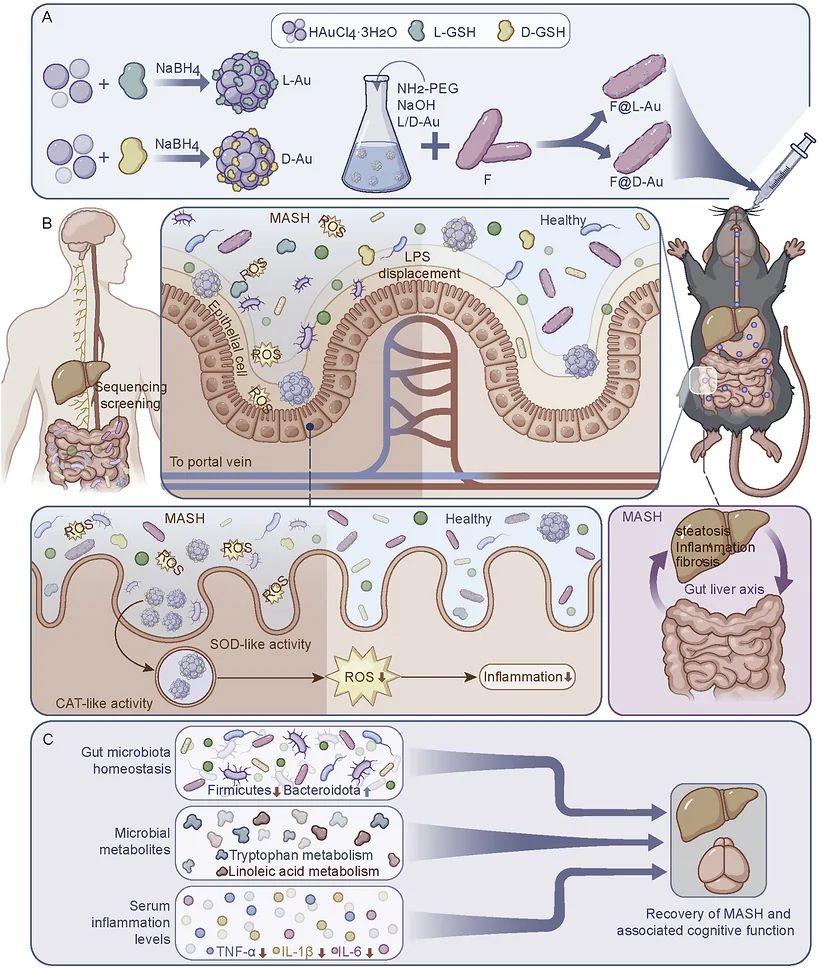
Schematic illustration of the design and therapeutic mechanism of F@D‐Au in MASH and associated cognitive impairment. (A) L‐Au and D‐Au nanozymes are prepared with L‐GSH and D‐GSH modification, followed by covalent conjugation to *F. duncaniae* to construct F@L‐Au and F@D‐Au. (B) Left: Human cohort screening. 16S rDNA sequencing of fecal samples from healthy controls and MASH patients identifies *F. duncaniae* as a key differential bacterium. Right: Au nanozymes scavenge gastrointestinal ROS via CAT‐ and SOD‐mimicking activities. Nanozymes bound to the surface of *F. duncaniae* protect bacteria against gastrointestinal stress and support their persistence within the intestine. F@D‐Au preserves intestinal barrier function, reduces portal LPS levels, attenuates pathological gut–liver axis signaling, and ameliorates hepatic lesions. (C) F@D‐Au remodels gut microbiota homeostasis, regulates microbial metabolism, and reduces proinflammatory mediators, ultimately alleviating MASH and associated cognitive impairment.

Orally administered F@L‐Au/F@D‐Au targets the gut–liver axis. The nanozyme moieties exert CAT‐ and SOD‐mimicking activities to scavenge ROS within the gastrointestinal tract. Nanozymes bound to the surface of *F. duncaniae* protect bacteria against harsh gastrointestinal conditions, enabling the bacteria to survive and persist within the intestine. F@L‐Au/F@D‐Au alleviate oxidative injury of intestinal epithelium and restore intestinal barrier integrity. This lowers portal LPS concentrations, attenuating pathological gut–liver axis signaling and ameliorating hepatic steatosis, inflammation, and fibrosis (Figure 9B).

F@D‐Au intervention remodels gut microbiota homeostasis, restores the *Firmicutes*/ *Bacteroidota* balance, modulates microbial tryptophan and linoleic acid metabolism, and reduces proinflammatory mediators in liver, hippocampus, and serum. These multiple gut‐centered beneficial effects collectively ameliorate MASH and its associated cognitive impairment (Figure 9C).

In this study, we demonstrated the therapeutic potential of a novel chiral‐engineered probiotic platform (F@D‐Au) through integrated modulation of the gut–liver axis for MASH treatment. These results suggest a promising integrated strategy that simultaneously targets hepatic and cognitive complications, meriting further evaluation in clinical trials.

## 3 Discussion

Our results indicate that the use of nanomedicine or probiotics alone is insufficient, as probiotics are vulnerable to degradation by gastrointestinal fluids, leading to their inactivation. Artificial enzymes, which mimic the body's natural antioxidant defense systems, offer a potential alternative to conventional anti‐inflammatory drugs by scavenging ROS to reduce inflammation. In this study, we developed a novel microbial nanomedicine, F@D‐Au, by anchoring chiral gold nanozymes onto the surface of *F. duncaniae*. This design enables nano‐catalytic immunotherapy through the activation of bacterial metabolism and enhancement of enzymatic activity. Thanks to the protective properties of the nanozymes, the probiotics were able to withstand gastrointestinal fluid damage, successfully colonize the gut, and maintain prolonged retention.

A thorough investigation into the efficacy of this microbiota‐based nanomedicine was performed using a murine model of MASH. The results demonstrated that F@D‐Au was more effective than F@L‐Au in slowing MASH progression. F@D‐Au significantly improved intestinal barrier integrity, thereby inhibiting disease development. In mouse and rat models, administration of F@D‐Au markedly attenuated hallmark features of MASH—including hepatic steatosis, necroinflammation, ballooning degeneration, and fibrosis. In addition, this intervention enhanced both hepatic and circulating lipid parameters and led to a reduction in biomarkers indicative of liver injury. These results indicate that F@D‐Au, when administered under conditions of CDHFD induction, shows significant promise as a nanotherapeutic approach for treating MASH.

To investigate the underlying mechanisms of F@D‐Au's protective effects against MASH, we hypothesized that its efficacy may stem from modulation of the gut microbiota and associated metabolome, thereby protecting hepatocytes from injury. Examination of the intestinal microbiome revealed that administration of F@D‐Au elevated the proportion of *Bacteroidota* while reducing *Firmicutes*, ultimately altering the microbial community structure to approximate that observed in healthy control animals. Oral administration of F@D‐Au improved gut dysbiosis in MASH mice, providing new insights into potential mechanisms for disease mitigation.

Subsequent metabolomic analysis revealed alterations in fecal tryptophan‐ and serum linoleic‐acid‐related metabolic profiles following F@D‐Au treatment. Compared with the MASH group, F@D‐Au intervention was accompanied by elevated fecal kynurenic acid and melatonin, as well as increased serum phosphatidylcholine and linoleic acid. These metabolic shifts were correlated with the amelioration of MASH‐related hepatic phenotypes and the secondary improvement of MASH‐associated cognitive impairments. Moreover, the changes in serum linoleic acid were also correlated with reduced serum cholesterol and modulated blood‐glucose levels. Collectively, our metabolomic data indicated that multiple metabolic pathways were perturbed upon F@D‐Au administration, and these metabolic signatures were associated with the observed therapeutic outcomes.

In conclusion, microbial nanomedicine presents a promising approach for treating MASH. Compared to traditional pharmaceutical therapies, it offers several significant advantages, including sustained catalytic activity through cascades and enhanced biosafety. The approach and methodology presented in this study may offer valuable perspectives for future therapeutic development targeting metabolic disorders.

## 4 Limitations

The present study demonstrated that D‐Au exhibited superior SOD‐ and CAT‐mimicking antioxidant activities compared with L‐Au, and such enantiomer‐dependent ROS‐scavenging capacity contributed to prominent biological protective effects. Nevertheless, this study did not perform density functional theory simulations or high‐resolution surface characterization to clarify the definitive atomistic stereochemical mechanism underlying the higher catalytic activity of D‐Au. The core innovation and research scope of this work focus on the biological synergistic effects and translational potential of engineered probiotic nanozymes in remodeling the gut–liver axis and treating MASH. Detailed physicochemical simulation and atomic‐level mechanistic interpretation are beyond the scope of the current study. Further structural modeling and high‐precision characterization are required in future investigations to fully elucidate the stereospecific catalytic mechanism of chiral nanozymes.

This study has several limitations that should be acknowledged. The 16S rRNA sequencing and untargeted metabolomic analyses in this work were only performed on the control, MASH, and F@D‐Au groups, without parallel multi‐omics profiling of the individual F‐only group and free D‐Au group. Although comprehensive phenotypic evaluations in Figures 5, 6, and 8 have clearly verified that F@D‐Au exhibits superior therapeutic efficacy compared with native *F. duncaniae*, free D‐Au nanozyme, and their physical mixture, the current omics data cannot fully distinguish the independent molecular contributions of the probiotic strain and nanozyme component. Previous –studies have demonstrated that *F. duncaniae* actively regulates intestinal tryptophan metabolism and produces immunomodulatory indole metabolites [56, 57]. Consistent with these reports, our metabolomic results indicated marked reprogramming of tryptophan metabolism after F@D‐Au intervention. Accordingly, we speculate that the synergistic protective effects of F@D‐Au are closely associated with the remodeled intestinal tryptophan metabolic pathway, which represents a reasonable mechanistic interpretation based on the available evidence.

## 5 Methods

### 5.1 Ethical Issues

All animal experimental protocols were approved by the Laboratory Animal Center of Shanghai Tenth People's Hospital (approval number: SHDSYY‐2024‐10152), and all animal procedures were performed in accordance with national guidelines for laboratory animal research. For human‐derived samples, this study was approved by the Ethics Committee of Zhongshan Hospital, Fudan University (approval number: B2020‐085R). Written informed consent was obtained from all individual donors before sample collection. The study was conducted in accordance with the Declaration of Helsinki.

### 5.2 Materials and Reagents

*Faecalibacterium duncaniae* (DSM 17677ᵀ, formerly misclassified as *Faecalibacterium prausnitzii* A2‐165) was obtained from the Leibniz‐Institute DSMZ‐German Collection of Microorganisms and Cell Cultures, Germany. The strain was cultured anaerobically at 37°C for 2–3 days and is extremely oxygen‐sensitive. Sequence‐based identification information: whole‐genome shotgun sequence accession NZ_CP022479; 16S rRNA gene accession AJ270469.

GSH (≥98%) was obtained from GL Biochem., Ltd. (Shanghai, China), and L‐GSH (≥98%) was sourced from the same supplier. Chloroauric acid trihydrate (HAuCl_4_·3H_2_O), sodium borohydride (NaBH_4_), and N‐hydroxysuccinimide (NHS) were supplied by Macklin Inc. (Shanghai, China). Simulated gastric fluid and artificial intestinal juice were acquired from Regene Biotechnology Co., Ltd. (Beijing, China). The sulfo‐Cy3 NHS ester dye was purchased from Shanghai Aladdin Biochemical Technology Co., Ltd. Furthermore, assay kits for CAT, total SOD, malondialdehyde (MDA), hydroxyproline (HYP), and non‐esterified fatty acids (NEFA) were all sourced from Nanjing Jiancheng Bioengineering Institute (Nanjing, China). Cell Counting Kit‐8 (CCK‐8) assay kits, DCFH‐DA reactive oxygen species detection kits, and DNA damage detection kits (γ‐H2AX immunofluorescence method) were obtained from Beyotime Biotechnology (China). LPS (TLR4‐specific agonist) was obtained from Beyotime Biotechnology (Shanghai, China; Product No. S1732), supplied as a pre‐dissolved aqueous stock solution (2 mg/mL). TNF‐ α, IL‐1b, IL‐6, and assay kits were purchased from Wuhan Abebio Science Co., Ltd. (Wuhan, China). Mouse C‐reactive protein (CRP) assay kits and mouse insulin (INS) ELISA kits were acquired from Shanghai Meixuan Biological Science & Technology Ltd. (Shanghai, China).

### 5.3 Materials Characterization Methods

TEM and Elemental Mapping: A 3 µL sample was placed on a carbon‐coated 250‐mesh copper grid for analysis. The microstructure and composition of the nanosystem were observed using an FEI Tecnai G2 F20 microscope operating at 200 kV, along with JEOL equipment. Nanoparticle size analysis was conducted using Nano Measurer 1.2 software. Zeta Potential: Measurements were carried out using the Zetasizer Nanoseries (ZTS1240, Malvern Panalytical Ltd.). Biological Microscopy: Fluorescent sections were examined using a Nikon biological microscope (CI‐L). CLSM: Imaging and analysis were performed with a Zeiss LSM900 microscope. CD Spectroscopy: CD spectra were obtained in aqueous solution using a Jasco J1500 spectropolarimeter.

### 5.4 Material Preparation Methods

#### 5.4.1 Synthesis of L‐a‐Au or D‐Au

L‐Au or D‐Au was synthesized using a modified method adapted from Kumar et al. [20]. In brief, an aqueous solution of HAuCl_4_·3H_2_O was mixed with 5 mL of a freshly prepared aqueous solution of L‐ or D‐glutathione (L‐ or D‐GSH, 0.12 mmol). This mixture was then diluted in 36 mL of water. After continuously stirring for 30 min, a 2 mL solution of NaBH_4_ (0.88 mmol) in water was slowly added while maintaining rapid stirring to ensure the complete reduction of gold ions. The solution was continuously stirred for another two h, during which time its color gradually changed to brown. The final product underwent ultrafiltration at 10 620 × g for 20 min, followed by three washes with deionized water to remove any unbound GSH molecules.

#### 5.4.2 Preparation of F@L‐Au and F@D‐Au

To prepare F@L‐Au and F@D‐Au, 500 mg of PEG‐NH_2_ was dissolved in 10 mL of an aqueous solution containing 119 mg of L‐Au/D‐Au. After complete dissolution of PEG‐NH_2_, 0.5 mL of NaOH solution (240 mg mL^−1^) was added, and the mixture was allowed to react for 30 min. Following this, 2 mL of ascorbic acid (AA) solution (0.1 M) was added, and the mixture was stirred for an additional 30 min. The resulting solution was washed multiple times with deionized water and set aside for further use. For the conjugation step, 1 mL of PEG‐Au was mixed with a bacterial suspension containing 1.15 mg of EDC and 1.3 mg of NHS. The reaction mixture was stirred for 30 min, followed by centrifugation at 3000 rpm for 5 min to isolate bacteria conjugated with nanoparticles. The conjugated bacteria were then washed three times with PBS.

### 5.5 Enzyme‐like Activity of L‐Au or D‐Au

#### 5.5.1 SOD Activity Assay for L‐Au or D‐Au

For the detection of SOD activity, 20 µL of each material was taken and analyzed using the WST‐1 method, strictly following the instructions provided in the kit. The absorbance was measured at 450 nm using OD readings. These measurements were documented to assess SOD activity, which was subsequently quantified based on the obtained absorbance values.

#### 5.5.2 CAT Activity Assay for L‐Au or D‐Au

The CAT activity was assessed by monitoring the decomposition of hydrogen peroxide (H_2_O_2_). To immediately terminate the reaction, ammonium molybdate was introduced, enabling it to interact with any residual H_2_O_2_ and generate a light‐yellow complex. The absorbance at 405 nm was then recorded, which facilitated the determination of CAT enzymatic activity.

### 5.6 Cellular Experiment Methods

#### 5.6.1 Cytotoxicity Assessment

The cytotoxic effects of L‐Au and D‐Au nanoparticles were evaluated utilizing the Cell Counting Kit‐8 (CCK‐8) assay. Briefly, HT29 cells were seeded in 96‐well plates at a density of 5 × 10^4^ cells/mL, with each well containing 100µL of complete culture medium. Cells were allowed to adhere by incubating at 37°C for 24 h. Subsequently, the medium was aspirated and replaced with 100µL of fresh medium supplemented with different concentrations of L‐Au or D‐Au (ranging from 0 to 100 µg /mL; final concentrations: 0, 3.125, 6.25, 12.5, 25, 50, and 100 µg /mL). After an additional 24‐h exposure to the nanoparticles, the cells were gently rinsed twice with pre‐warmed phosphate‐buffered saline (PBS) to remove residual particles and medium. Next, 100 µL of new medium containing 10µL of CCK‐8 reagent was dispensed into each well, followed by incubation for another 4 h at 37°C. The optical density was subsequently measured at 450 nm using a microplate reader to assess cell viability. Each experimental condition was performed in quadruplicate (*n* = 4).

#### 5.6.2 Cellular Uptake Assessment

The cellular uptake of L‐Au and D‐Au was evaluated using Cy3 dye. Cells were seeded onto coverslips and cultured in a 12‐well plate to allow adherence. Then, culture medium containing Cy3‐labeled L‐Au or D‐Au was added, and the cells were incubated at 37°C in the dark for various durations (0, 0.5, 2, 4, and 6 h). After incubation, the cells were washed three times with PBS. To observe the cellular uptake process, the nuclei were stained with DAPI, and CLSM was used for visualization.

#### 5.6.3 Intracellular ROS Detection

Intracellular ROS levels were quantified using a ROS assay kit that utilizes the oxidation‐sensitive fluorescent dye DCFH‐DA, following the manufacturer's guidelines. Intestinal epithelial cells were first pre‐treated with LPS (1 µg/mL) for 12 h and then categorized into four experimental groups: control, LPS, LPS + L‐Au (L‐Au = 100 µg/mL), and LPS + D‐Au (D‐Au = 100 µg/mL). Following a 12‐h co‐incubation period, cells were treated with 1 mL of serum‐free medium containing 10 µM DCFH‐DA and maintained at 37°C for 20 min. Subsequently, any excess DCFH‐DA was eliminated by washing the cells three times with serum‐free medium. ROS generation was then quantified via flow cytometry and visualized through fluorescence microscopy.

#### 5.6.4 Protein Carbonylation Assessment

Protein carbonylation levels were assessed using a 2,4‐dinitrophenylhydrazine (DNPH) derivatization method.

#### 5.6.5 Lipid Peroxidation Assessment

Lipid peroxidation was assessed using the C11‐BODIPY581/591 fluorescent dye.

#### 5.6.6 DNA Damage Assessment

The cells were first fixed with 500µL of 4% paraformaldehyde at room temperature for 20 min. After fixation, the cells were washed with PBS and then permeabilized with 500 µL of 0.5% Triton X‐100. Subsequently, the cells were blocked with 1 mL of 1% BSA/PBS solution for 1 h to minimize non‐specific binding. Next, the cells were incubated overnight at 4°C with a mouse monoclonal anti‐phospho‐histone H2AX antibody diluted at 1:500. After incubation, an Alexa Fluor 488‐conjugated secondary antibody (diluted 1:500 in 1% BSA/PBS) was applied in the dark for 1 h. The cells were then washed with PBS to remove any unbound antibodies, followed by staining the nuclei with 10 µM DAPI for 5 min. Finally, DNA damage was detected and analyzed by CLSM.

#### 5.6.7 ELISA for Inflammatory Cytokines

The concentrations of TNF‐a, IL‐1β, and IL‐6 were measured using ELISA.

#### 5.6.8 Simulated Gastric Fluid Experiment (SGF)

*F. duncaniae*, F@L‐Au, F@D‐Au, F+L‐Au, and F+D‐Au were co‐cultured with SGF at 37°C for 4 h. The proliferation of engineered bacteria in SGF was evaluated via spectrophotometric OD measurements at 0, 1, 2, 3, and 4 h.

#### 5.6.9 Simulated Intestinal Fluid Experiment (SIF)

*F. duncaniae*, F@L‐Au, F@D‐Au, F+L‐Au, and F+D‐Au were co‐cultured with SIF at 37°C for 10 h. The growth of the engineered bacteria in SIF was assessed by measuring the OD using a spectrophotometer at 0, 2, 4, 6, 8, and 10 h.

### 5.7 Animal Experiments

#### 5.7.1 Animal Subjects

Male SD rats and C57BL/6J mice, both six weeks of age, were purchased from Beijing Vito Biological Technology Co., Ltd. The animals were maintained under specific pathogen‐free (SPF) conditions with a 12‐h light/dark schedule, ambient temperatures kept at 25°C, and relative humidity maintained between 40% and 70%. Animals were provided ad libitum access to water. To induce a MASH model, rodents were fed a CDHFD for a period of 16 weeks.

#### 5.7.2 In Vivo Safety Assessment

Male six‐week‐old C57BL/6J mice were randomly assigned to eight groups (*n* = 3 per group). Each cohort received oral gavage of either L‐Au or D‐Au at concentrations of PBS, 5 mg/kg, 10 mg/kg, or 15 mg/kg. Blood was collected from the animals for comprehensive hematological and serum biochemical assessments. After the dosing regimen, major organs—including the heart, liver, spleen, lungs, kidneys, stomach, intestines, and brain—were excised and processed for histopathological analysis using H&E staining.

#### 5.7.3 Quantification of *F. duncaniae* by Quantitative PCR

The absolute abundance of *F. duncaniae* was determined by qPCR using species‐specific primers targeting *F. duncaniae* (FaecaliF: 5′‐AAGGGCCGCGGTTATGTGCCT‐3′; FaecaliR: 5′‐TAATCGATGGCCTGTCCAACG‐3′), designed in the present study [58]. qPCR assays were performed in duplicate in a final volume of 10 µL containing 5 µL of 2× SYBR Green master mix, 3 µL of nuclease‐free water, 1 µL of primer mix (5 µM each of forward and reverse primer), and 1 µL of template DNA. Amplification was carried out under the following cycling conditions: initial denaturation at 95°C for 5 min, followed by 40 cycles of denaturation at 95°C for 20 s, annealing at 63°C for 25 s, and extension at 72°C for 30 s. Melting curve analysis was subsequently performed: 95°C for 15 s, 60°C for 30 s, followed by a gradual temperature ramp from 60°C to 95°C to verify amplification specificity. Absolute quantification was achieved by comparing sample cycle threshold (Ct) values against a standard curve generated from ten‐fold serial dilutions of genomic DNA extracted from *F. duncaniae*.

#### 5.7.4 Intraperitoneal Glucose Tolerance Test

Mice underwent an overnight fast of approximately 16 h while being allowed unrestricted access to water. Subsequently, each mouse received an intraperitoneal injection of a 10% glucose solution at a dose of 1 g/kg body weight. Blood glucose concentrations were determined using a glucometer (Yuwell, Cat. No. 680 cr) by sampling from the tail vein at 0, 15, 30, 60, and 120 min after glucose administration. To reduce the risk of excessive bleeding, the injection site was momentarily cleansed with 75% ethanol. Following completion of the test, animals were placed in fresh cages, provided with food and water, and monitored for at least one h post‐procedure.

#### 5.7.5 Intraperitoneal Insulin Tolerance Test

The injection site was cleaned with 75% ethanol wipes to minimize bleeding, as done in the glucose tolerance test.

#### 5.7.6 Evaluation of Liver Lipid Peroxidation

To assess hepatic lipid peroxidation, levels of TBARS were quantified. Initially, a 10% liver homogenate (45 µL) was mixed with 675 µL of 20% acetic acid, 675 µL of 0.8% thiobarbituric acid solution (purchased from Sigma–Aldrich), and 405 µL of deionized water to prepare the reaction mixture. This blend was incubated for 60 min in a water bath maintained at 95°C. After heating, 600 µL of the reaction product was combined with 750 µL of an n‐butanol/pyridine mixture (15:1, v/v) as well as 150 µL of distilled water. The resulting solution was vigorously vortexed, followed by centrifugation to separate the phases. The absorbance of the upper organic layer was determined at 532 nm using a spectrophotometer. A standard curve was established using serial dilutions of 1,1,3,3‐tetramethoxypropane to facilitate quantification.

#### 5.7.7 Hydroxyproline Measurement

Hydroxyproline (HYP) in liver tissue was quantified using an HYP determination kit according to the manufacturer's standard protocol.

#### 5.7.8 Malondialdehyde (MDA) Measurement

MDA in liver tissue was assessed using an MDA testing kit based on standard methods.

#### 5.7.9 Morris Water Maze (MWM) Test

C57BL/6J mice/SD rats were divided into five groups: Control, MASH, D‐Au, F, and F@D‐Au groups. All experimental animals were male. SD rats and C57 mice were trained and assessed in a water maze with diameters of 1.6 meters and 1.2 meters, respectively. A set of stationary visual cues was placed on the maze walls to aid in navigation. Mice trained for 4 days (C57) and 5 days (SD) received three acquisition trials each day. Following the training phase, a probe trial was performed to evaluate spatial memory.

#### 5.7.10 New Object Recognition (NOR) Test

This study primarily aimed to evaluate the recognition ability of mice toward new objects, following a three‐step experimental protocol: First, mice were familiarized with an open field environment for 5 min each day over 3 consecutive days. Second, two identical objects regarding size and material were placed in the familiarized open field, allowing mice to explore for 5 min. Third, after 3 h, one of the objects was replaced with a novel object that had a similar height but a completely different shape. Exploration times for both new and familiar objects were recorded over 5 min. After each experiment, the open field and objects were disinfected with alcohol to eliminate odors, and the entire experimental process was conducted in a closed and quiet environment.

#### 5.7.11 In Vivo Imaging Experiment

The *F. duncaniae* bacterial cells were first incubated with IR‐783 for 1 h, followed by centrifugation at 3000 rpm for 5 min. The pellet was washed three times with bacterial broth to remove unbound excess dye. The IR‐783‐labeled F bacteria were then covalently conjugated with L‐Au or D‐Au nanozymes to obtain F@L‐Au and F@D‐Au, respectively. Control and MASH group mice received an oral gavage of 300 µL of F, F@L‐Au, or F@D‐Au suspension. In vivo fluorescence imaging was performed to trace the colonization of the engineered bacteria. 24 h after administration, mice were euthanized, and major organs including the heart, liver, spleen, lungs, kidneys, brain, and gastrointestinal tract were harvested for ex vivo imaging.

#### 5.7.12 HE and Sirius Red Staining

Tissue samples were initially fixed in 4% paraformaldehyde overnight. Following fixation, they underwent sequential washes with methanol, ethanol, a 1:1 mixture of ethanol and xylene, and finally pure xylene, prior to paraffin embedding. The paraffin‐embedded samples were cut into sections 5 µm thick and subsequently stained with hematoxylin for 10 min and Sirius Red for 1 h. After the staining process, slides were washed twice with acidified water, dehydrated through a series of 100% ethanol and xylene baths, and mounted using DPX mounting medium (Sigma).

#### 5.7.13 Oil Red O Staining

After fixation, the sections were stained with a 60% Oil Red O working solution for 15 min to assess lipid accumulation and counterstained with hematoxylin for 2 min to visualize cell structures. After staining, cover slips were applied using melted glycerin gelatin (Sigma–Aldrich), which was kept at 50°C to ensure proper mounting. The slides were then examined using a microscope (Olympus IX83).

#### 5.7.14 Blood–Brain Barrier Integrity Evaluation

Blood–brain barrier permeability was assessed via the Evans blue extravasation assay. Briefly, mice were injected with 2% Evans blue solution through the tail vein at a dosage of 4 mL/kg. After 120 min of circulation, the animals were anesthetized and subjected to transcardial perfusion with pre‐cooled PBS until the effluent became clear. Whole brain tissues were isolated, weighed, and homogenized in formamide, followed by incubation in the dark to extract extravasated Evans blue. The supernatant absorbance was measured at 620 nm using a microplate reader. The brain Evans blue content was calculated based on the standard curve.

#### 5.7.15 Fecal and Serum Metabolomics Analysis

Fecal specimens were collected in sterile tubes, promptly frozen, and maintained at −80°C to ensure the integrity of metabolites. Whole blood samples were held at ambient temperature for 2 h to facilitate clotting. The samples were then centrifuged at speeds of 2000–3000 rpm for 10 min to obtain serum, which was immediately snap‐frozen and stored at −80°C for later analysis. Metabolites were separated and identified using liquid chromatography–mass spectrometry (LC‐MS).

### 5.8 Statistical Analysis

All statistical analyses were performed using GraphPad Prism 9.3.1. The normality of continuous variables was evaluated before statistical testing. The unpaired t‐test was applied for normally‐distributed two‐group data, while the Mann‐Whitney U test was used for datasets that did not follow a Gaussian distribution. For multiple‐group comparisons, one‐way ANOVA followed by Tukey's multiple comparisons test was used. Statistical significance was defined as *p* < 0.05. Significance symbols: ns, not significant (*p* > 0.05), ^*^ *p* < 0.05, ^**^ *p* < 0.01, ^***^ *p* < 0.001, ^****^ *p* < 0.0001.

### 5.9 Human Cohort and Diagnostic Criteria

All participants underwent conventional B‐mode abdominal ultrasound and serum liver function tests. For MASH participants, liver fibrosis stage and NAS components (steatosis, lobular inflammation, and hepatocellular ballooning) were assessed. All MASH subjects received histopathological diagnosis via liver biopsy. Fibrosis stages and NAS scores were determined based on liver histopathology. Individuals with excessive alcohol intake, viral hepatitis, autoimmune liver disease, or drug‐induced liver injury were excluded from this study. Healthy controls exhibited normal liver biochemistry and no hepatic steatosis on abdominal ultrasound; liver biopsy was not performed in healthy participants.

## Author Contributions

**Jing Chen**: conceptualization, investigation, writing – original draft, visualization, validation, methodology, software, supervision, writing – review and editing. **Shen Zhang**: conceptualization, methodology, validation, software. **Yuli Zhu**: investigation, software, methodology, writing – original draft. **Da Zhou**: validation, methodology, software. **Chunya Ji**: validation, methodology. **Mingrui Zhu**: investigation, software. **Sirui Wang**: formal analysis, writing – review and editing. **Yuting Shen**: methodology. **Jifeng Yu**: software, data curation. **Heng Zhang**: methodology, writing – review and editing, supervision. **Yan Fang**: supervision, data curation, software, methodology. **Huixiong Xu**: conceptualization, writing – review and editing, funding acquisition, supervision. **Haohao Yin**: project administration, resources, funding acquisition, writing – review and editing, conceptualization, supervision, data curation.

## Funding

This work was supported by the National Natural Science Foundation of China (grants 82430064 to H.X.X., 82302206 to H.H.Y., and 82102050 to Y.F.), the National Key Research and Development Program of the Ministry of Science and Technology of China (grants 2023YFC2414204, 2023YFC2414203, and 2023YFC2414202 to H.X.X.), the Shanghai Municipal Health Commission (SHSLCZDZK 03502 and 202340087), the Scientific Research and Development Fund of Zhongshan Hospital, Fudan University (2022ZSQD07, 2024ZSQN24, and 2003‐002), the Shanghai Sailing Program (23YF1441600), and the China Postdoctoral Science Foundation (2023T00073).

## Conflicts of Interest

The authors declare no conflicts of interest.

## Supporting information




**Supporting File**: advs78432‐sup‐0001‐SuppMat.docx.

## Data Availability

The data that support the findings of this study are available from the corresponding author upon reasonable request.
