# Supplementary material for: Engineered Chiral Probiotics Ameliorate MASH via Gut–Liver Axis
Source: Adv Sci (Weinh). 2026 Nov 4:e78432. Online ahead of print. doi: 10.1002/advs.78432 (PMC13736141; doi:10.1002/advs.78432)
Supplement: Supplementary file 1 [file ADVS-9999-e78432-s001.docx]

**Engineered Chiral Probiotics Ameliorate MASH via Gut–Liver Axis**

Jing Chen^1,2†^, Shen Zhang^1†^, Yuli Zhu^1†^, Da Zhou^3†^, Chunya Ji^1^, Mingrui Zhu^1^, Sirui Wang^1^, Yuting Shen^1^, Jifeng Yu^1^, Heng Zhang^2*^, Yan Fang^4*^, Huixiong Xu^1*^and Haohao Yin^1*^

*^1.^Department of Ultrasound, Zhongshan Hospital, Institute of Ultrasound in Medicine and Engineering, Fudan University, Shanghai 200032, P. R. China.*

*^2.^ Department of Ultrasound, Zhuhai People's Hospital (The Affiliated Hospital of Beijing Institute of Technology, Zhuhai Clinical Medical College of Jinan University)，Zhuhai 519000, China.*

*^3.^ Department of Gastroenterology, Zhongshan Hospital, Fudan University, Shanghai 200032, China*

*^4.^ Department of Ultrasound, Huashan Hospital, Fudan University, Shanghai 200040, China.*

***Corresponding Author:***

*Huixiong Xu: xuhuixiong@126.com, Department of Ultrasound, Zhongshan Hospital. Institute of Ultrasound in Medicine and Engineering, Fudan University, Shanghai 200032, No.180 Fenglin Road, China*

*Haohao Yin: yin.haohao@zs-hospital.sh.cn, Department of Ultrasound, Zhongshan Hospital. Institute of Ultrasound in Medicine and Engineering, Fudan University, Shanghai 200032, No.180 Fenglin Road, China*

*Yan Fang: almostspring@126.com, Department of Ultrasound, Huashan Hospital, Fudan University, Shanghai, 200040, No. 12 Urumqi Middle Road, China*

*Heng Zhang: www616www@126.com, Department of Ultrasound, Zhuhai People's Hospital (The Affiliated Hospital of Beijing Institute of Technology, Zhuhai Clinical Medical College of Jinan University)，Zhuhai 519000, China.*

**Figure S1.** Zeta potential of L-Au, D-Au, F, F@L-Au, and F@D-Au (n = 3 independent samples).

**Figure S2.** Simulated enzyme activity and ROS scavenging ability of L-Au and D-Au. (A) SOD-like activity of L-Au and D-Au at different concentrations (n = 3 independent samples). (B) CAT-like activity of L-Au and D-Au at different concentrations (n = 3 independent samples). Comparisons between groups were performed using two‑way analysis of variance (two‑way ANOVA), followed by Sidak’s multiple‑comparisons test for pairwise comparisons between L‑Au and D‑Au at each concentration. Data are presented as mean ± standard deviation. ns, not significant (p>0.05), *p<0.05, **p<0.01, ***p<0.001, ****p<0.0001.

**Figure S3.** ROS scavenging capability of different materials on LPS-induced intestinal epithelial cells under oxidative stress, using DCFH-DA as the ROS probe, observed via fluorescence microscopy. The concentration of LPS stimulation was 1μg/mL for 12 hours, and the treatment concentrations of L-Au and D-Au were 100μg/mL. (A) Fluorescence microscopy images were captured following various treatment conditions. Representative images from three independent replicates within each experimental group are presented. The scale bar represents 200μm. (B) Corresponding mean fluorescence intensity. Comparisons among multiple groups were conducted using one‑way analysis of variance (one‑way ANOVA), followed by Tukey’s multiple‑comparisons post‑hoc test (B). Data are presented as mean ± standard deviation. ns, not significant (p>0.05), *p<0.05, **p<0.01, ***p<0.001, ****p<0.0001.

**Figure S4.** Animal Safety Assessment (300μL per animal). (A) In vivo hematological parameters. Hematological analysis of healthy mice treated with different doses of L-Au (PBS, 5mg/kg, 10mg/kg, 15mg/kg). Each group consisted of three biologically independent samples (n = 3). (B) Tissue sections from healthy mice treated with varying doses of L-Au were subjected to H&E staining (PBS, 5mg/kg, 10mg/kg, 15mg/kg). Representative images from three replicates in each group are shown. (C) In vivo hematological parameters. Hematological analysis of healthy mice treated with different doses of D-Au (PBS, 5mg/kg, 10mg/kg, 15mg/kg) Each group consisted of three biologically independent samples (n = 3). (D) Tissue sections from healthy mice, which were treated with varying doses of D-Au, underwent H&E staining (PBS, 5mg/kg, 10mg/kg, 15mg/kg). Representative images from three replicates in each group are shown. Comparisons among multiple groups were conducted using one‑way analysis of variance (one‑way ANOVA), followed by Tukey’s multiple‑comparisons post‑hoc test (A, C). Data are presented as mean ± standard deviation. ns, not significant (p>0.05).

**Figure S5.** OD values of F, F@L-Au and F@D-Au before and after conjugation with IR783 dye. Grouping: F, F+IR783, F@L-Au, F@L-Au+IR783, F@D-Au, F@D-Au+IR783 (n = 3). Comparisons among multiple groups were conducted using one‑way analysis of variance (one‑way ANOVA), followed by Tukey’s multiple‑comparisons post‑hoc test. Data are presented as mean ± standard deviation. ns, not significant (p>0.05).

**Figure S6.** Biodistribution of gold nanozymes and *F.duncaniae* in mouse organs 24 h after intragastric gavage. (A) ICP‑MS quantification of gold concentration in mouse liver 24 h after intragastric gavage. Grouping: MASH+F, MASH+F@L-Au, MASH+F@D-Au. (n = 3). (B)Absolute qPCR analysis of *F.duncaniae* gene copies in colonic mucosa 24 h after intragastric gavage. Gene copies were log₁₀‑transformed for visualization. Grouping: Control, MASH, MASH+F, MASH+F@L-Au, MASH+F@D-Au. (n = 3). Comparisons among multiple groups were performed using one‑way analysis of variance (one‑way ANOVA), followed by Tukey’s multiple‑comparisons post‑hoc test. Data are presented as mean ± standard deviation. ns, not significant (p>0.05), *p<0.05, **p<0.01, ***p<0.001, ****p<0.0001.

**Figure S7.** Changes in blood glucose levels in MASH mice after treatment. Grouping: Control, MASH, L-Au, D-Au, F, F@L-Au, F@D-Au. (A) Serum Glu (n = 5). (B) Serum INS (n = 5). (C, E) GTT (n = 5) and (D, F) ITT (n = 5). Comparisons of time-dependent curves in GTT and ITT were performed using two-way analysis of variance (two-way ANOVA), followed by Sidak’s multiple‑comparisons test for post‑hoc pairwise comparisons (C, D). Comparisons among multiple groups were performed using one‑way analysis of variance (one‑way ANOVA), followed by Tukey’s multiple‑comparisons post‑hoc test (A, B, E, F). Data are presented as mean ± standard deviation. ns, not significant (p>0.05), *p<0.05, **p<0.01, ***p<0.001, ****p<0.0001.

**Figure S8.** Changes in liver and body weight in MASH mice after treatment. Grouping: Control, MASH, L-Au, D-Au, F, F@L-Au, F@D-Au. (A) Macroscopic appearance and representative morphological images of the liver from mice in different treatment groups. (B) Liver weight, body weight, and liver-to-body weight ratio (n = 10). Comparisons among multiple groups were performed using one‑way analysis of variance (one‑way ANOVA), followed by Tukey’s multiple‑comparisons post‑hoc test. Data are presented as mean ± standard deviation. ns, not significant (p>0.05), *p<0.05, **p<0.01, ***p<0.001, ****p<0.0001.

**Figure S9.** Changes in blood biochemical parameters and inflammation cytokine levels in MASH mice after treatment. Grouping: Control, MASH, L-Au, D-Au, F, F@L-Au, F@D-Au. (A) Serum concentrations of TG, TC, FFA, LDL, and HDL were measured (n = 5). (B) Serum levels of CRP were quantified (n = 5). (C) Serum concentrations of TNF-α, IL-1β, and IL-6 were determined (n = 5). Comparisons among multiple groups were performed using one‑way analysis of variance (one‑way ANOVA), followed by Tukey’s multiple‑comparisons post‑hoc test. Data are presented as mean ± standard deviation. ns, not significant (p>0.05), *p<0.05, **p<0.01, ***p<0.001, ****p<0.0001.

**Figure S10.** Hepatic biochemical analysis and levels of fibrosis and oxidative stress in MASH mice after treatment. Grouping: Control, MASH, L-Au, D-Au, F, F@L-Au, F@D-Au. (A) Hepatic tissue levels of TC and TG (n = 5). (B) Hepatic hydroxyproline content (n = 5). (C) Levels of hepatic MDA and TBARs (n = 5). Comparisons among multiple groups were performed using one‑way analysis of variance (one‑way ANOVA), followed by Tukey’s multiple‑comparisons post‑hoc test. Data are presented as mean ± standard deviation. ns, not significant (p>0.05), *p<0.05, **p<0.01, ***p<0.001, ****p<0.0001.

**Figure S11.**F@D-Au Significantly Improves Cognitive Function in MASH Mice. Grouping: Control (n = 10), MASH (n = 10), D-Au (n = 10), F (n = 10), F@D-Au (n = 10). (A) Crossing number of the target quadrant during the Morris water maze test. (B) Dwell time in the target quadrant during the Morris water maze test.(C) Escape latency to the target quadrant during the Morris water maze test. Each "n" represents an independent biological sample. Comparisons among multiple groups were performed using one‑way analysis of variance (one‑way ANOVA), followed by Tukey’s multiple‑comparisons post‑hoc test. Data are presented as mean ± SEM. ns, not significant (P>0.05), *P<0.05, **P<0.01, ***P<0.001, ****P<0.0001.

**Figure S12.**F@D-Au ameliorates the increased permeability of the blood–brain barrier. Grouping: Control (n = 5), MASH (n = 5), D-Au (n = 5), F (n = 5), F@D-Au (n = 5). Representative images and quantitative analysis of Evans blue leakage (A) and serum S100β levels (B). Comparisons among multiple groups were performed using one‑way analysis of variance (one‑way ANOVA), followed by Tukey’s multiple‑comparisons post‑hoc test. Data are presented as mean ± standard deviation. ns, not significant (p>0.05), *p<0.05, **p<0.01, ***p<0.001, ****p<0.0001.

**Figure S13.** F@D-Au Improves Cognitive Function in MASH Mice. Grouping: Control, MASH, D-Au, F, F@D-Au. (A) Representative images of GFAP expression in the hippocampus detected by immunofluorescence staining. (B) Representative images of MAP2 expression in the hippocampus detected by immunofluorescence staining. Scale bars represent 100μm in both panels.

**Figure S14.** Illustration of the impact of oral F@D-Au administration on α-diversity in MASH mice (n = 6). (A) Chao1 index across different groups. (B) Shannon index for each group. (C) Simpson index among various groups. Each "n" represents an independent biological sample. Comparisons among multiple groups were performed using one‑way analysis of variance (one‑way ANOVA), followed by Tukey’s multiple‑comparisons post‑hoc test. Data are presented as mean ± standard deviation. ns, not significant (p>0.05), *p<0.05, **p<0.01, ***p<0.001, ****p<0.0001.

 **Figure S15.** Heatmap visualizations display marked changes in gut microbial composition at the phylum (A) and genus (B) classifications, comparing the Control, MASH, and F@D-Au experimental groups. These comparative analyses highlight the distinct microbial profiles associated with each treatment condition.

**Figure S16.** Illustration of serum metabolomics analysis (n = 6). (A) PLS-DA of serum metabolite profiles was conducted, with the 95% confidence intervals for each group represented by ellipses. (B) The volcano plot demonstrates the differential serum metabolites identified when comparing the F@D-Au and MASH groups. Metabolites that are significantly increased in the experimental group are marked in red, whereas those that are significantly decreased are shown in blue. Metabolites without statistically significant differences between the two groups are depicted as gray points. (C) A heatmap is presented to display the significant alterations in serum metabolites across the Control, MASH, and F@D-Au groups, focusing on the top 50 most differential metabolites. (D) A bubble chart is presented, illustrating the metabolic pathways that are significantly enriched with differential metabolites between the F@D-Au and MASH groups. (E and F) Variations in essential metabolites involved in the linoleic acid metabolic pathway were examined when comparing the treatment group to the MASH group. These differences highlight key metabolic shifts associated with linoleic acid metabolism between the two experimental conditions. Comparisons between the two groups were performed using the unpaired t‑test (E, F). Data are presented as mean ± standard deviation. ns, not significant (p>0.05), *p<0.05, **p<0.01, ***p<0.001, ****p<0.0001.

**Figure S17.** Representative ultrasound images of rat livers from the Control, MASH, and F@D‑Au groups (n = 3 biological replicates per group).

**Figure S18.** Liver tissues collected from SD rats subjected to various interventions underwent staining procedures including H&E, Masson’s Trichrome, Sirius Red, and Oil Red O. The figure displays representative microscopic images each selected from three independent biological replicates per experimental group. The scale bar corresponds to 100 µm.

| **Characteristic** | **Healthy controls (n=7)** | **MASH group (n=9)** | **Statistic** | **P-value** |
| --- | --- | --- | --- | --- |
| Age (years) | 51.43±17.27 | 48.33±11.25 | t=0.43 | 0.671 |
| Height (cm) | 164.71±5.02 | 165.22±9.47 | t=−0.13 | 0.900 |
| Weight (kg) | 61.43±8.28 | 76.41±12.09 | t=−2.80 | 0.014 |
| BMI (kg/m²) | 22.56±2.08 | 27.91±3.12 | t=−3.90 | 0.002 |
| ALT (U/L) | 21.71±10.09 | 139.00±43.97 | t=−7.74 | <0.001 |
| AST (U/L) | 24.14±8.28 | 98.89±32.93 | t=−6.55 | <0.001 |
| Triglycerides (mmol/L) | 0.96±0.39 | 1.86±0.42 | t=−4.43 | <0.001 |
| Total cholesterol (mmol/L) | 4.08±0.49 | 5.20±0.37 | t=−5.26 | <0.001 |
| Sex (Male/Female) | Male 3 (42.9%),  Female 4 (57.1%) | Male 3 (33.3%),  Female 6 (66.7%) | Fisher | 1.000 |
| Medication | None: 7 (100.0%) | None: 9 (100.0%) | - | - |
| Liver fibrosis stage | F0: 7(100.0%) | F1: 5(55.6%), F2: 2(22.2%), F3: 1(11.1%), F4: 1(11.1%) | U=0 | <0.001 |
| Steatosis (NAS, 0–3) | 0: 7(100.0%) | 1: 1 (11.1%), 2: 4 (44.4%), 3: 4 (44.4%) | U=0 | <0.001 |
| Lobular inflammation (NAS, 0–3) | 0: 7(100.0%) | 1: 1 (11.1%), 2: 4 (44.4%), 3: 4 (44.4%) | U=0 | <0.001 |
| Hepatocellular ballooning (NAS, 0–2) | 0: 7(100.0%) | 1: 7(77.8%), 2: 2(22.2%) | U=0 | <0.001 |
| MASH score (NAS total, 0–8) | 0: 7(100.0%) | 4: 1 (11.1%), 5: 4 (44.4%), 7: 3 (33.3%), 8: 1 (11.1%) | U=0 | <0.001 |

**Supplementary Table 1.** Baseline clinical characteristics of MASH patients and healthy controls in the pilot study. Continuous variables are presented as mean ± standard deviation, and categorical variables as n (%). Sex was compared using Fisher's exact test.
